# A New Reference Genome Assembly for the Microcrustacean *Daphnia pulex*

**DOI:** 10.1534/g3.116.038638

**Published:** 2017-02-22

**Authors:** Zhiqiang Ye, Sen Xu, Ken Spitze, Jana Asselman, Xiaoqian Jiang, Matthew S. Ackerman, Jacqueline Lopez, Brent Harker, R. Taylor Raborn, W. Kelley Thomas, Jordan Ramsdell, Michael E. Pfrender, Michael Lynch

**Affiliations:** * Department of Biology, Indiana University, Bloomington, Indiana 47405; † Department of Biology, University of Texas at Arlington, Texas 76019; ‡ Laboratory for Environmental Toxicology, Ghent University, B-9000, Belgium; § Notre Dame Genomics and Bioinformatics Core Facility, University of Notre Dame, Indiana 46556; ** School of Informatics and Computing, Indiana University, Bloomington, Indiana 47405; †† Hubbard Center for Genome Studies, University of New Hampshire, Durham, New Hampshire 03824; ‡‡ Department of Biological Sciences, University of Notre Dame, Indiana 46556; §§ Environmental Change Initiative, University of Notre Dame, Indiana 46556

**Keywords:** genome annotation, effective population size, gene number, intron, mobile elements

## Abstract

Comparing genomes of closely related genotypes from populations with distinct demographic histories can help reveal the impact of effective population size on genome evolution. For this purpose, we present a high quality genome assembly of *Daphnia pulex* (PA42), and compare this with the first sequenced genome of this species (TCO), which was derived from an isolate from a population with >90% reduction in nucleotide diversity. PA42 has numerous similarities to TCO at the gene level, with an average amino acid sequence identity of 98.8 and >60% of orthologous proteins identical. Nonetheless, there is a highly elevated number of genes in the TCO genome annotation, with ∼7000 excess genes appearing to be false positives. This view is supported by the high GC content, lack of introns, and short length of these suspicious gene annotations. Consistent with the view that reduced effective population size can facilitate the accumulation of slightly deleterious genomic features, we observe more proliferation of transposable elements (TEs) and a higher frequency of gained introns in the TCO genome.

The ultimate goal of comparative genomics is to form a synthesis integrating the fundamental evolutionary forces explaining the variation of genomic architecture across a wide range of phylogenetic lineages. The reduced effective population size (*N*_e_) of eukaryotic species relative to prokaryotic species is hypothesized to be centrally involved in the emergence and distribution of numerous genomic features unique to eukaryotes, and the associated principles should naturally extend to variation among lineages of closely related species (Lynch 2007). However, until recently, it has been difficult to study the impact of reduced *N*_e_ on the evolution of genomic architecture because of the lack of genomic data from closely related species or populations with disparate population sizes. Moreover, a lack of parallel understanding of other fundamental microevolutionary parameters such as mutation and recombination rates complicates efforts to single out the role of *N*_e_ (and the associated power of random genetic drift) on genome evolution.

Here, we focus on the comparative genomics of two cyclically parthenogenetic *Daphnia pulex* clones (PA42 and TCO), which come from populations differing substantially in historical *N*_e_. The *D. pulex* species complex consists of a vast array of populations inhabiting hundreds of thousands of ponds and lakes, throughout the northern temperate zone, most of which (including PA42 and TCO) reproduce by repeated generations of clonal reproduction via unfertilized eggs, punctuated by a phase of sexual reproduction. PA42 was sampled from a woodland vernal pond within Portland Arch Nature Preserve, IN, whereas TCO was derived from a permanent pond in the Siuslaw National Forest, near the Pacific coast in OR. A complex geological history in western OR contributes to the presence of divergent lineages of multiple zooplankton species (Dyke *et al.* 2002; Shafer *et al.* 2010), including *D. pulex* (Crease *et al.* 1997). The lineage containing TCO is thought to have diverged from midwest US *D. pulex* as a consequence of geographic isolation during Pleistocene cycles of glaciation, and OR *D. pulex* is sometimes assigned a distinct species designation, *D. arenata*. However, the level of nucleotide sequence divergence between TCO and *D. pulex* in the Midwestern US is only twice that of the average within-population divergence of alleles in the latter (Lynch *et al.* 2016).

Consistent with the glacial relict hypothesis, the level of nucleotide diversity within the population from which TCO is derived is <10% of that found in typical midwest populations, including the source population of PA42 (Lynch *et al.* 1999; Omilian and Lynch 2009). Given the similar estimates of the base-substitution mutation rate (*u*) derived from mutation-accumulation experiments applied to these two lineages (Keith *et al.* 2016), and given that silent-site diversity estimates the composite quantity 4*N*_e_*u*, we estimate that the TCO source population has a *N*_e_ ∼ 10% of that of typical populations in the midwest US.

The substantially reduced heterozygosity in the TCO isolate served the practical purpose of avoiding the technical difficulties that can arise when assembling a highly heterozygous genome, and hence led to the first *Daphnia* genome sequencing project (Colbourne *et al.* 2011). The resultant TCO assembly and annotation implied a surprisingly large number of genes (∼31,000), greatly exceeding the numbers in most other arthropod genomes and including a large number of recent gene duplicates. Remarkably, an unusually high rate of intron gain is exhibited by the TCO genome, and more generally by OR *D. pulex*, relative to all other metazoans and arthropods (Colbourne *et al.* 2011; Li *et al.* 2009, 2014). Concerns have been raised about a potential overestimation of the gene number in the TCO genome, resulting from assembly issues such as the fragmentation of genes into different contigs, one argument being that ∼20% of the gene annotations in the original TCO assembly lack translation initiation and/or termination codons (Denton *et al.* 2014). However, no draft genome assembly is without problems, and it is plausible that many of the aberrant features of the TCO genome may reflect real evolutionary responses to a long-term reduction in *N*_e_ (Lynch 2007). Here, we present a *de novo* assembly of the genome of the PA42 isolate of *D. pulex*, constructed using a combination of paired-end, mate-pair libraries, and synthetic long reads (SLRs), with annotation guidance provided by RNA-sequencing (RNA-seq) data from animals grown under diverse environmental conditions. Although not perfect, this assembly is of considerably higher quality than the previous one reported for TCO, is likely much more representative of the genomic architecture of the study species, and yields insights into the genome-wide consequences of reduced effective population size.

## Materials and Methods

### Daphnia culture

The *D. pulex* isolate PA42 was obtained from a woodland vernal pond, Portland Arch (latitude: 40.2013, longitude: −87.3294), IN in May 2013. This isolate is cyclically parthenogenetic, *i.e.*, reproduces asexually in favorable environmental conditions and sexual reproduction is triggered by deteriorating conditions, such as high population density and food shortage. This isolate was maintained under favorable laboratory conditions so that they could reproduce parthenogenetically, and was fed *ad libitum* with a suspension of the algae *Scenedesmus obliquus*.

### De novo genomic assembly

We assembled the genome of the *D. pulex* PA42 isolate *de novo*. DNA of the PA42 isolate was extracted from mass culture of clonally reproducing individuals. To minimize bacterial contamination, for any DNA collected here for sequencing libraries we starved and treated the culture for 2 d with 50 mg/L ampicillin and 50 mg/L streptomycin libraries in COMBO media. We made paired-end sequencing libraries with insert sizes of 350, 450, and 600 bp using the NEBNext Ultra DNA Library Prep Kit for Illumina (New England Biolabs, Ipswich, MA). Also, we made mate-pair libraries with insert sizes of 3, 5, 11, 19, and 27 kbp using Nextera Mate Pair Sample Prep Kit (Illumina, Inc., San Diego, CA) (Supplemental Material, Table S1 in File S4). The libraries were sequenced on a HiSequation 2500 sequencing platform (Illumina) with 250 bp paired-end reads. Low quality regions and sequencing adapter contamination were trimmed from the raw reads using Trimmomatic (Bolger *et al.* 2014) with parameters: ILLUMINACLIP:True-seq:2:30:10 HEADCROP:3 SLIDINGWINDOW:4:15 MINLEN:30. The adapter sequences on mate-pair sequences were removed using the software Nextclip with default parameters (Leggett *et al.* 2014). We used AllPathsLG version 43460 (Gnerre *et al.* 2011) for generating genomic assembly. The parameter for running PrepareAllPathsInputs.pl are “PLOIDY = 2 GENOME_SIZE = 200,000,000 FRAG_COVERAGE = 45 JUMP_COVERAGE = 45 LONG_JUMP_COVERAGE = 1 PICARD_TOOLS_DIR = picard-tools-1.52.” Parameters for RunAllPathsLG are “HAPLOIDIFY = True REFERENCE_NAME = PA42 TARGETS = standard EVALUATION = BASIC.” Then we generated SLRs to connect the scaffolds further and remove the contaminants. We prepared a 10 kbp library using a TruSeq Synthetic Long-Read DNA Library Prep Kit (P/N FC-126-1001, Illumina, Inc.) according to the TruSeq Synthetic Long-Read DNA Library Prep Guide (P/N 15047264 Rev. B, Illumina, Inc.) with BluePippin Size-selection (TruSeq Synthetic Long-Read DNA Library Prep Guide Supporting Material). High quality gDNA (500 ng) was sheared by g-Tube (P/N 520079, Covaris, Woburn, MA). End repair, dA-tailing, and adaptor ligation was performed on sheared gDNA. The ligated products went through a BluePippin Size-selection protocol for 8 k–10 kbp range and were quantified with qPCR. Based on qPCR results, the library was diluted and distributed into a 384-well plate, whereby each well contained ∼6–8 fg of the size-selected library, for long range PCR. Next, the library went through Nextera tagmentation with 384 different barcoding PCR primers. Final products were pooled, concentrated, and were dual-sided size-selected with Sample Purification beads. Bioanalyzer was used for final validation of average size and absence of primer contamination. A single pool library was sequenced on HiSequation 2500 Rapid Run mode with 150 bp paired-end reads, producing 483 million reads. TruSeq adapter sequences were trimmed using Trimmomatic (Bolger *et al.* 2014) in paired-end mode. Error correction and assembly of filtered reads was completed using the multi-cell SPADES 3.5.0 pipeline (Nurk *et al.* 2013) with default settings. Contigs larger than 8000 bp were used for further analysis. First, SLRs were used for rescaffolding the draft assembly PA42 1.0 using SSPACE-long reads software (Boetzer and Pirovano 2014). Then, we used PBjelly (English *et al.* 2012) to fill the gaps; the result was PA42 2.0.

Because the presence of bacteria in *Daphnia* culture media and/or possible endosymbiont species (Qi *et al.* 2009) can introduce foreign DNA sequences into the final assembly, we took several measures to remove bacterial DNA contamination. First, we blasted all the scaffolds against NCBI’s nonredundant nucleotide database (NR) to remove scaffolds with bacteria origin. Scaffolds with *e* value < 1e−5 and >95% identity to bacterial sequences were removed. Second, we used an independently derived paired-end sequencing library of PA42 (PE data) and a collection of 104 single sperm whole-genome sequence data to verify true *Daphnia* scaffolds. These two datasets were mapped to PA42 scaffolds using bwa-mem (version 0.7.2) with default parameters (Li and Durbin 2009), then we calculated the breadth of coverage for each scaffold (nucleotide bases in a scaffold that were covered by sequencing reads). In total, scaffolds summing up to 150 million bases of the PA42 genome have >80% breadth of coverage by both PE and sperm reads (Figure S1 in File S4), strongly suggesting their noncontaminant status. We manually checked the scaffolds with high PE read coverage (>80%) but lower sperm read coverage (<80%) by blasting them against the NR; the results show that most of their top hits were *Daphnia* sequences in TCO except for scaffolds with <30% breadth of sperm read coverage. Given that whole-genome amplification was performed before sequencing single sperm, which is likely to introduce bias in recovering different genomic regions, we decided to keep scaffolds with >80% breadth of coverage from PE reads and >30% from sperm read coverage in the final assembly.

### Gene prediction and functional annotation

We annotated the PA42 draft genome using three different strategies, *i.e.*, RNA-seq-guided gene discovery, protein homology, and *ab initio* gene prediction. To perform annotation of the PA42 draft assembly using empirical gene expression data, we generated a set of RNA-seq libraries for the PA42 isolate (Table S2 in File S4) under three different life stages (mature males, adult parthenogenetic female, and adult presexual reproduction female) and seven different environmental conditions (*i.e.*, treatments by temperature, Atrazine, UV light, pH, NaCl, and Nickel). The transcriptome for each life stage/condition was assembled *de novo* using the software Trinity (Grabherr *et al.* 2011), with the pooled, total transcriptome consisting of 177,598 transcripts. These transcripts were then used as seed for evidence-based gene annotation using the software Maker version 2.0 (Holt and Yandell 2011). A total of 18,257 genes were found based on RNA-seq-guided annotation. Proteins from the Reference Species (Ecdysozoans *Caenorhabditis elegans*, *Strigamia maritime*, *Anopheles gambiae*, and *Drosophila melanogaster*, and *Homo sapiens*) together with 458 proteins from CEGMA (Parra *et al.* 2007) were used as input for MAKER (Holt and Yandell 2011) to identify genes with homologous evidence; in total, 19,830 genes were identified. Furthermore, we performed *ab initio* gene prediction on the PA42 draft assembly using the software SNAP (Korf 2004) trained by 800 genes with complete gene structure (*i.e.*, possessing 3′-UTR, 5′-UTR, and more than three exons) that are supported by RNA-seq. The *ab initio* annotation generated a set of 18,990 genes. As these three annotation strategies generated overlapping gene predictions, we used a conservative strategy to include genes with at least two pieces of evidence. Our gene annotation resulted in a final set of 18,440 genes (Figure S2 in File S4). We used the Benchmarking set of Universal Single-Copy Orthologs (BUSCO) (version 1.2; Simão *et al.* 2015) to estimate the completeness of our genome. The metazoans database for BUSCO was downloaded from http://busco.ezlab.org/, then all the proteins predicted in our analysis were used to blast the metazoan database in BUSCO with -trans parameter, and the rest of the parameters were set as default. All the predicted proteins were blasted against NCBI’s NR using blastp program with an *e*-value set to 1.0e−3. A total of 16,806 genes (91%) were assigned with a corresponding gene name after this NR blast. We searched motifs and domains against public databases, including Pfam, PRINTS, PROSITE, ProDom, SMART, PANTHER, Gene3D, HMMpham, and SuperFamily through InterProScan 5.14 (Jones *et al.* 2014). Gene Ontology (GO) IDs and terms for each gene were assigned based on InterPro results. We also mapped the reference genes to KEGG and Enzyme codes. Of the genes, 4570 had an enzyme code and could be assigned to 117 KEGG pathways. The annotation process was done using blast2go to select GO terms generated by blasting to InterPro, the parameters we used were *E*-value = 1.0E−6, annotation cutoff = 55, and GO-weight = 5. Then, ANNEX (Götz *et al.* 2008) was used to correct the annotation. Annex can add and correct biological processes and cellular component GOs based on molecular function GOs.

### Identification of TEs

Three classes of full-length TEs, including LTR retrotransposons, non-LTR retrotransposons, and DNA transposons, were *de novo* identified in the PA42 reference genome, respectively. Two computer softwares, LTRharvest (Ellinghaus *et al.* 2008) and MGEScan (Rho *et al.* 2007), were used to identify all potential intact LTR retrotransposons. We further filter the candidate LTR TEs based on similarity (>80%) and the lengths (>80 bp), as well as the presence of putative *Pol* ORFs. The EMBOSS package (Olson 2002) was used to search against Pfam HMM files (Finn *et al.* 2014) to identify key protein domains of *Pol* ORFs (*E*-value < 1e−10). Reverse transcriptase (RT) sequences of each element were extracted to conduct phylogenetic analysis using Mafft (Katoh *et al.* 2005) and FastTree (Price *et al.* 2009) to get the subclass information. Non-LTR retrotransposons were identified using MGEScan-nonLTR (Rho and Tang 2009). Similar to the procedure of classifying LTR elements into different subclades, RT sequences were extracted and used to classify different non-LTR elements. DNA transposons were identified by using program MGEScan-DT (W. M. Ismail and H. Tang, unpublished data), which searches for genes encoding transposases in the genome by HMMer and extends on both sides of the hit region to locate the TIRs. This program automatically classifies the DNA elements into different clades.

All subclasses of TEs were combined and removed redundancy with full sequence similarity >98% by CD-HIT (Fu *et al.* 2012), constituting a nonredundant full-length TE library. This library was used in a homology search to detect all the fragmented TEs in the PA42 reference genome using RepeatMasker (Smit *et al.* 2004). Only the elements with the best score in the overlapping ones with a length of at least 500 bp were kept in our analysis.

### Relative rate test

To evaluate the differences of molecular evolution between the PA42 and TCO genome comparisons, we performed the relative-rate test (Sarich and Wilson 1967; Wu and Li 1985) to compare the substitution rates for silent and replacement sites, *K*_s_ and *K*_a_, respectively, using sequences of *D. obtusa*, a closely related species, as an outgroup (Tucker *et al.* 2013).

To calculate *K*_s_ and *K*_a_ in PA42/*D. obtusa* and TCO/*D. obtusa*, we mapped *D. obtusa* reads to the PA42 and TCO genome assemblies using bwa-mem (version 0.7.2) (Li and Durbin 2009), respectively. Reads with multiple mapping were excluded and bases in the reference genome with <4 *D. obtusa* reads covered were not used in further analyses. Then, the consensus sequence for *D. obtusa* was generated using SAMtools (Li *et al.* 2009). Next, we extracted the coding sequence of each gene in TCO and PA42 based on the TCO annotation file (Colbourne *et al.* 2011) and the PA42 annotation generated in this study. *K*_a_ and *K*_s_ for each gene were then calculated using yn00 from the PAML4.8a package (Yang 2007). The yn00 program implements several counting methods for estimating *K*_a_ and *K*_s_ between two sequences; here, we picked the results using realistic evolutionary models (Yang and Nielsen 2000).

### Gene family analysis

TCO, PA42, and Reference Species’ protein sequences were used in this analysis. Genes from TCO that may generated due to false annotations were removed prior to the analysis. For those genes with multiple transcripts, we retained the longest one, translated proteins were put into OrthoMCL (Li *et al.* 2003) to identify orthologs among Reference Species. Gene clusters were generated by OrthoMCL standard protocol. Genes in the orthologous clusters were assigned to Structural Classification of Proteins (SCOP) families (Murzin *et al.* 1995; Gough *et al.* 2001). Each SCOP family contains gene domains descended from a common ancestor. To identify the expansion and contraction of gene families we used CAFÉ 3.0 (Han *et al.* 2013) which relies on phylogenetic history to provide a statistical foundation for evolutionary inferences. To generate the species tree required by CAFÉ 3.0, single-copy gene families, which have one gene in each species, were extracted for phylogenic tree construction. Protein sequences of each gene were aligned using PRANK (Löytynoja and Goldman 2005) and then concatenated using custom Perl scripts (File S3). Maximum Likelihood trees were constructed using MEGA6 (Tamura *et al.* 2013). The RelTime-ML program implemented in the MEGA6 package was used to estimate divergence time among species and calibration time was obtained from the TimeTree database (http://www.timetree.org/).

### Data availability

TCO sequences and gene models used in this analysis were downloaded from JGI (http://genome.jgi.doe.gov/Dappu1/Dappu1.download.ftp.html). The gene model we used is FrozenGeneCatalog20110204; proteins and coding sequences used are FrozenGeneCatalog20110204.proteins.fasta and FrozenGeneCatalog20110204.CDS.fasta.gz, respectively. *D. magna* data were downloaded from wfleabase (http://server7.wfleabase.org/genome/Daphnia_magna/openaccess/genome/altassembly/), the draft assembly used is nwbdmag24g7d_asm.fasta.gz, and the *D. magna* proteins used in this analysis are arp7s10b14nodmag.aa.gz. The Reference Species’ coding and protein sequences were downloaded from Ensembl (Ensembl 80: May 2015). The raw reads for this study are available at the Short Read Archive with the study accession no. PRJNA307976. The *D. pulex* genomic assembly PA42 v3.0 is available at the European Molecular Biology Laboratory (EMBL) Nucleotide Sequencing Database or NCBI under the accession no. PRJEB14656. Sperm reads used in this study can be found with accession no. SRP058678. The sequence of *D. pulex* clones and *D. obtusa* from Tucker *et al.* (2013) are deposited under the accession nos. SAMN02252729–SAMN02252752 in the NCBI Sequence Read Archive. There was an error in NCBI server that the *D. obtusa* sequences were replaced by something else. Therefore, we redeposited the *D. obtusa* to European Nucleotide Archive under the accession no. PRJEB17737. 

## Results

### Genome assembly of PA42

We achieved a *de novo* assembly of the genome of a natural *D. pulex* isolate (PA42) using AllPathsLG (Gnerre *et al.* 2011) and a combination of paired-end and mate-pair Illumina sequencing libraries (650 × total coverage, Table S1 in File S4). An additional 52,443 SLRs (>8000 bp) were used to support the further connection of scaffolds and filling gaps. The draft genome presented here, PA42 3.0, consists of 156 Mb of DNA sequences located on 1822 scaffolds with a N50 scaffold size of 494 kb (Table S3 in File S4). The N50 scaffold number is 96 and the largest scaffold is 1,661,524 bp (Table 1). A genetic map based on whole-genome sequencing of 104 single sperm (Xu *et al.* 2015a) anchors 366 scaffolds into 12 chromosomes, constituting 86.6% of the draft assembly and leaving 13.4% of the physical map unassigned with respect to chromosomes (File S1).

**Table 1:** Summary of the genomic metrics for the PA42 and TCO genomes

	**TCO**	**PA42 3.0**
Genome size, including gaps (bp)	197,261,574	156,418,198
Number of scaffolds	5191	1822
Length of largest scaffold (bp)	4,163,030	1,661,524
Mean scaffold length (bp)	38,001	85,849
Number of N50 scaffold	75	96
Total length of gaps (bp)	38,612,943	13,454,790
Number of annotated genes	30,097	18,440
Mean length of a coding gene (including introns) (bp)	2289	2998
Mean number of exons/gene	6.6	6.9
Mean exon size (bp)	212	237
Mean intron size (bp)	169	223
Mean UTR size (bp)	371	214
Fraction of long introns	10%	14%

TCO data are compiled from Colbourne *et al.* (2011). Long introns are defined as having a length exceeding the average exon size. UTR, untranslated region.

To help achieve accurate annotation, we performed RNA-seq on PA42 individuals exposed to seven environmental conditions (low pH, UV exposure, salinity, etc.) and three male and female life stages (Table S3 in File S4). We predicted 18,440 genes using an annotation approach guided by RNA sequences in combination with protein homology and *ab initio* prediction with the software Maker 2.0 (Holt and Yandell 2011) (File S3 and Figure S2 in File S4). In line with the expectation for a high quality set of annotated genes, we find that the completeness of our genome assembly is likely to be very high based on comparison with a list of nearly universal arthropod genes (Waterhouse *et al.* 2013; Simão *et al.* 2015); of the subset of genes that are found in >90% of described arthropod genomes, we find 96% of these within the PA42 annotation list.

In the final annotation of protein-coding genes in PA42, 1535 (∼8%) lack a canonical translation initiation codon and/or a translation termination codon, with 4% lacking an ATG start codon, 5% lacking a stop, and 1% lacking both (Table 2). In contrast, there are 3395 (11.2%) annotated genes in the TCO genome lacking one or the other, and 1102 (3.6%) lacking both. Not all genes have canonical start codons, and the lack of an annotated stop codon can simply be a consequence of a lack of RNA-seq support in the downstream region of a gene. Thus, it is difficult to establish whether these are erroneously or simply incompletely annotated genes. For the 977 genes lacking stop codons in the PA42 annotation, 735 have RNA-seq covering >50% of the coding region, and 408 have identifiable homologs in a set of Reference Species (Ecdysozoans *C. elegans*, *S. maritime*, *A. gambiae*, and *D. melanogaster*, and *H. sapiens*) used for comparative analyses (described below) and TCO.

**Table 2:** Comparison of TCO and PA42 gene annotations

	**TCO**	**PA42 3.0**
Numbers of genes	30,097	18,440
With homologs in Reference Species*^a^*	17,062	13,260
*D. pulex*-specific genes*^b^*	5715	3818
Lineage specific genes*^c^*	7320	1362
Genes without canonical start codon	2278	798
Genes without stop codon	2219	977
Genes without start and stop codons	1102	244
Potential excess genes from gene split events*^d^*	190	62

a Reference Species: *C. elegans*, *S. maritime*, *A. gambiae*, *D. melanogaster*, and *H. sapiens*.

b Genes in PA42 or TCO with no identified Reference homologs, but that share homology with each other as identified by reciprocal blasts.

c Genes without identified homologs in either the Reference Species or the alternative *D. pulex* genome.

d Splitting of a single gene in one genome assembly into multiple pieces in the other assembly, with each piece annotated as a separate gene in the second assembly.

### Comparison of the PA42 and TCO annotations

**Figure 1:**
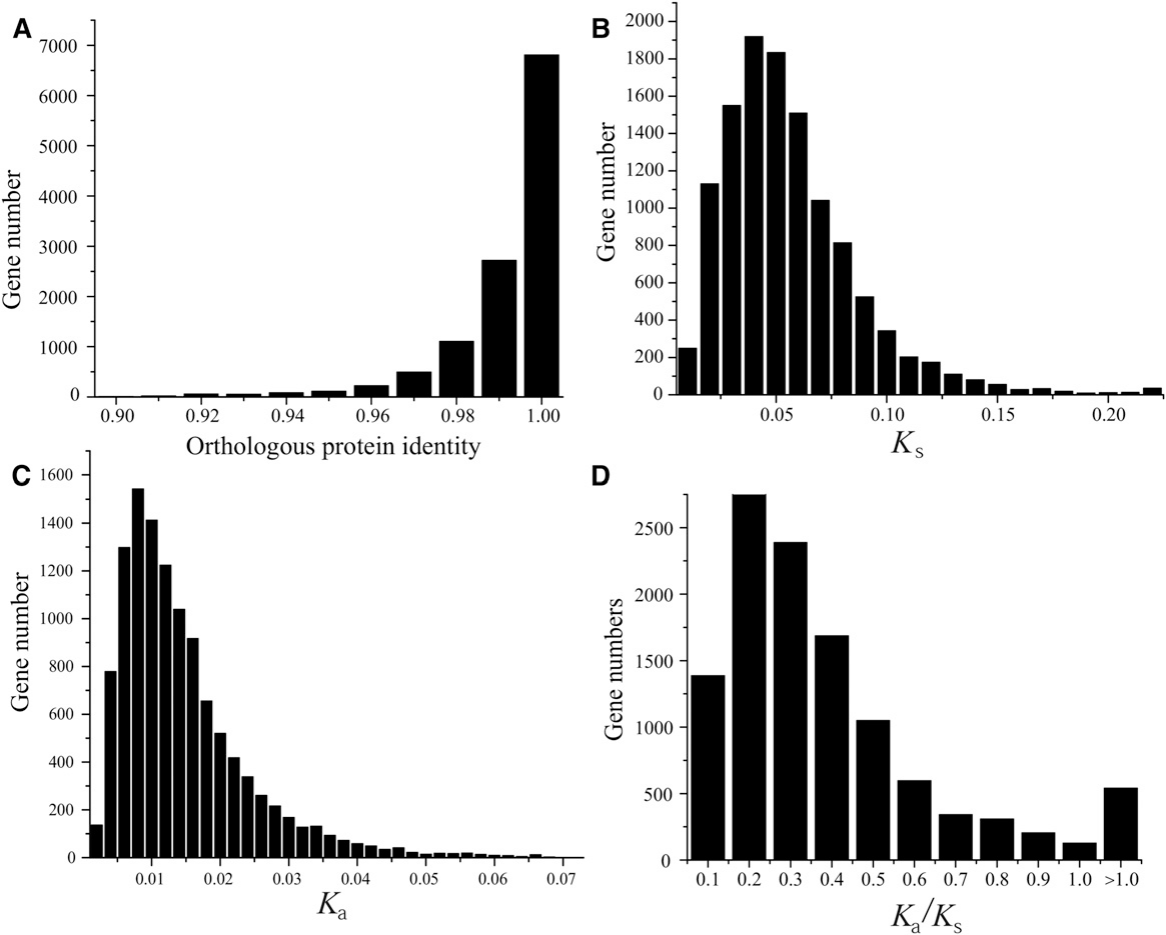
Distributions of the features of protein-coding sequences for the 11,694 one-to-one orthologs in TCO and PA42 (>300 bp alignment length). (A) Amino acid sequence identity. (B) Pairwise divergence at silent sites, *K*_s_. (C) Pairwise divergence at replacement sites, *K*_a_. (D) The ratio *K*_a_/*K*_s_.

To determine the sources of the substantially different numbers of annotated genes between PA42 and TCO (18,440 *vs.* 30,097), we performed comparative analyses of the two gene sets. A search of the annotated protein sequences of each gene in PA42 against the NR in NCBI reveals homologous sequences for 16,500 genes, 94% of which have best hits against members of the deposited TCO annotation (Figure S3 in File S4). Based on reciprocal-blast analysis, the number of one-to-one orthologous genes between TCO and PA42 is estimated to be 11,780, which we judge to be *bona fide D. pulex* genes. The total PA42 assembly displays larger average exon and intron sizes than those of TCO, with an average protein-coding gene (including introns) span of ∼3000 bp in PA42 *vs.* ∼2300 bp in TCO (Table 1). If this comparison is restricted to the set of 11,780 TCO-PA42 orthologs, the average lengths of coding exons and introns are ∼10% greater in PA42 than in TCO. However, this is primarily due to the presence of more introns and exons in the longest bin in PA42, as the size distributions are otherwise extremely similar, having the same modes (Figure S4 in File S4). The protein sequences of the 1:1 orthologs on average have 98.8% identity, with nearly 60% of them being 100% identical (Figure 1A). The average synonymous (*K*_s_) and nonsynonymous (*K*_a_) differences per nucleotide site are 0.0512 and 0.0136, respectively (Figure 1, B and C). The median *K*_a_/*K*_s_ ratio is 0.254 (Figure 1D), similar to that found in comparisons of the fruit fly *D. melanogaster* to other *Drosophila* species (Bergman *et al.* 2002).

Given the base-substitution mutation rate in *D. pulex*, 5.7 × 10^−9^/site/generation (Keith *et al.* 2016), under the assumption of neutrality, which appears to be fulfilled for silent sites in this species (Lynch *et al.* 2016), the observed average level of *K*_s_ implies that the average pair of TCO-PA42 orthologs diverged ∼4.8 million generations ago (or assuming five generations per year, ∼1 million years ago). To put this in perspective, analysis of a population near the PA locality yields an estimate of π_s_ = 0.0183 for the average within-population silent-site divergence between random alleles (Lynch *et al.* 2016). Because the population from which TCO was obtained is nearly completely inbred (with π_s_ ≈ 0.001; Colbourne *et al.* 2011), a corrected measure of silent-site divergence between the OR and IN *D. pulex* is 0.0512 − (0.0183/2) = 0.0421 (Nei and Li 1979), reducing the previous time estimates by 18%.

To investigate the potential phylogenetic source of the large difference in gene numbers between these two clones, we queried the protein sequences of TCO and PA42 against the gene lists for four other Ecdysozoans (*C. elegans*, *S. maritime*, *A. gambiae*, and *D. melanogaster*) and *H. sapiens*. Using an e-value cutoff of 0.01, we detected 17,062 genes in TCO and 13,260 in PA42 with homologs in these Reference Species (Table 2). For PA42 and TCO genes with no identified Reference homologs, reciprocal blasts between these two genomes identified an additional 3818 genes in PA42 and 5715 in TCO to have homologs across the two assemblies; these are potentially *D. pulex*-, *Daphnia*-, cladoceran-, or crustacean-specific genes. The remaining annotated genes in each genome without any identified metazoan homologs number 7320 in TCO and 1362 in PA42.

It has been argued that the TCO gene number estimate is inflated due to errors in genome assembly and/or annotation (Denton *et al.* 2014). To evaluate whether genome assembly and/or annotation error in TCO is responsible for the bulk of the inflated gene number, we performed several analyses. First, orthologous gene clusters were generated among PA42, TCO, and the above-noted Reference Species using OrthoMCL (Li *et al.* 2003). This program uses a Markov cluster algorithm to group orthologs and in-paralogs (genes duplicated after speciation) across multiple taxa. We found 13,375 orthologous clusters to contain genes from PA42 and/or TCO. In 68% of these cases, the gene number is identical for both clones, whereas 22% of the clusters have more genes from TCO and 10% have more genes from PA42. If divergent alleles were artificially assembled and annotated as paralogs in the TCO genome (but not in PA42), this might partially explain the elevated TCO gene numbers in orthologous clusters (with a 2× inflation expected for single genes that are erroneously annotated as two paralogs in TCO). For the orthologous clusters with more genes in TCO, 17% had a gene number >2× that in PA42, and in 5% the number of TCO genes was ≤2× that in PA42. In principle, the latter category could include some erroneous annotation of alleles as genes (although this is unlikely owing to the extreme homozygosity of TCO), but clusters with >2× inflation in gene number in TCO cannot be accommodated by such an explanation (unless the baseline gene number in a cluster in TCO also exceeds that in PA42).

Another potential reason for the gene number discrepancy between TCO and PA42 is the artifactual splitting of a single gene into multiple pieces (and each piece being enumerated as a separate gene in the TCO annotation). Such errors are expected to result in two or more genes in one annotation mapping to different regions of a single gene in the other annotation. To quantify the impact of such merge/split events, we reciprocally blasted the protein sequences of the two assemblies and detected more split events in TCO compared to PA42 (179 *vs.* 58, *P* < 0.0001, Table S4 in File S4), with most of them being two genes in one assembly merged into one gene in the other. A total of 369 genes in TCO were merged to 179 genes in PA42, and 120 genes in PA42 were merged into 58 genes in TCO. Without additional information from an alternative genome or mRNA sequencing data for these genes we are unable to resolve which annotation is correct; nonetheless, this overall analysis implies that gene fragmentation is a relatively minor issue in both the TCO and PA42 assemblies.

Looking more closely at the 17% of orthologous clusters with >2× genes in TCO relative to PA42 (8346 genes in total), we found that most of these lack detectable orthologs in other species (Table S5 in File S4). A total of 7320 such genes have no obvious orthologs in either PA42 or the Reference Species employed in this analysis, and of these TCO-specific genes, 631 are singletons, while the remaining 6689 form orthologous clusters of two or more genes. For this latter subset, the average pairwise *K*_S_ for paralogs is 0.050 ± 0.001, which is similar to the average pairwise *K*_S_ for the 1:1 TCO-PA42 orthologs (0.056 ± 0.001), implying recent duplication.

Possible explanations for this substantial number of TCO-specific genes are errors in annotation, artifacts of some sort of microbial contamination in the TCO DNA source, products of rampant horizontal gene transfer into the TCO genome (possibly of multiple origins), or a combination of these effects. However, the “orphan” TCO genes are highly scattered at the contig and scaffold levels in the TCO assembly, in 59% of cases having nearest neighbors with orthologs in PA42 (Table S6 in File S4), so it is unlikely that they are derived from misassembly associated with a contaminating bacterial genome sequence. Fortuitously, whole-genome sequence exists for several mutation-accumulation lines derived from TCO several years after the original genome sequencing project (Keith *et al.* 2016), and the raw reads from such clones map to 92% of the TCO orphans, providing some validation that much of the DNA associated with the TCO orphan genes is not a consequence of transient contamination. Genome-wide sequence is available for 11 other *D. pulex* clones derived from broadly separated geographic locations (including one from OR; Tucker *et al.* 2013), and we find that, on average, 11% of the reads from these clones map to TCO orphan gene DNA (Table S7 in File S4), covering the same orphans as in the PA42 analysis. Nonetheless, of the 7320 orphan annotated TCO genes: 592 are not covered by any read from TCO-derived mutation-accumulation lines and could involve some sort of contaminant; 575 exhibit homologous DNA sequence in PA42 but without shared flanking genes; and 2402 are not detected at the DNA level in PA42 (Table S6 in File S4).

Of the 4918 annotated TCO orphan genes with homologous DNA in PA42, 65% exhibit >95% nucleotide sequence identity and 85% exhibit >90% identity. Yet, despite this clear orthology, none of these TCO orphans are annotated in PA42; this is because 75% are without any mRNA support and 48% contain premature stop codons. To determine whether the subset of TCO orphans with orthologous DNA in other *D. pulex* are under selection (as would be expected for genes with functional significance), we estimated the average *K*_a_/*K*_s_ against PA42 and the 11 other *D. pulex* genomes. The distribution of average *K*_a_/*K*_s_ for these genes is quite broad, with a mean of 0.57, which is significantly greater than that for 1:1 TCO-PA42 orthologs (Figure S5 in File S4).

**Figure 2:**
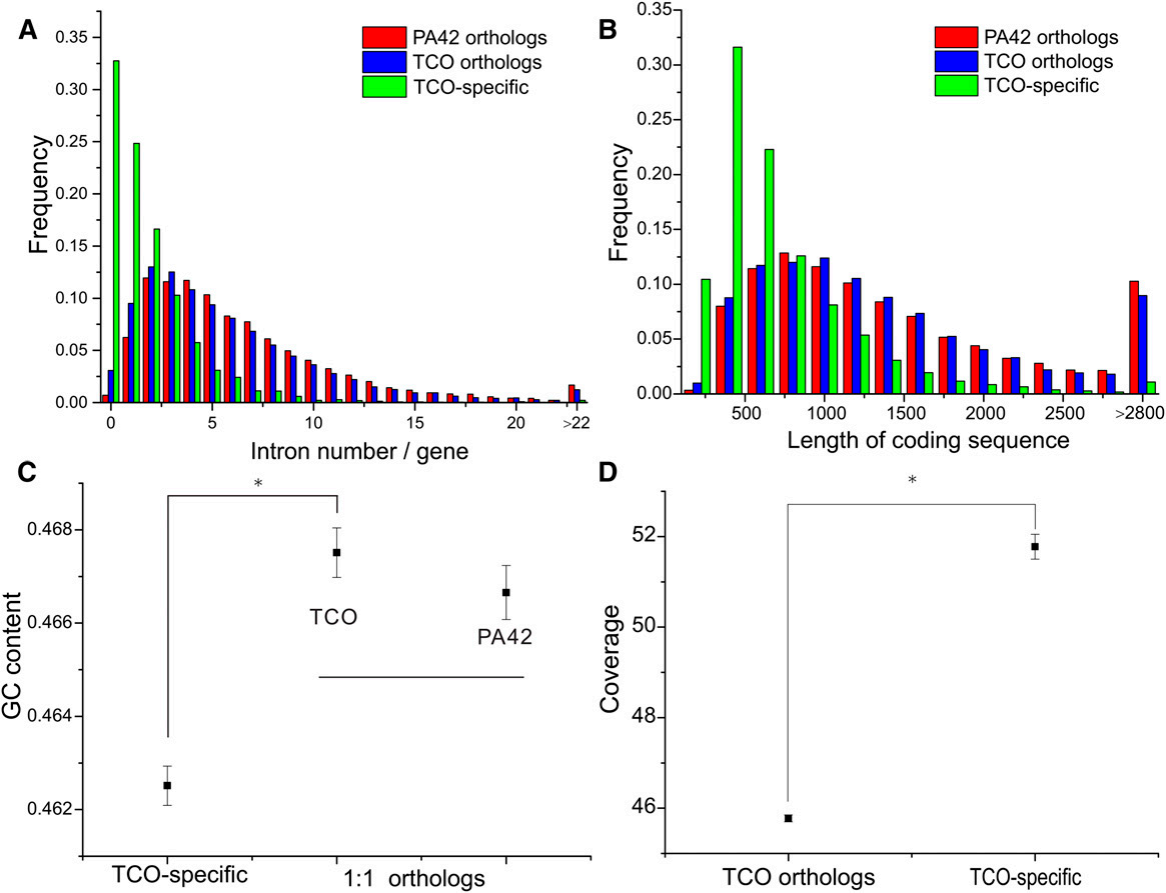
Comparison of the features of 1:1 TCO–PA42 orthologs and TCO-specific genes. TCO-specific genes have no obvious orthologs in PA42 or the Reference Species (other metazoans). (A) Distributions of intron numbers. (B) Length distributions for all coding sequences (excluding introns). (C) Comparisons of average GC contents. (D) Comparisons of average sequence coverages. Error bars indicate SEs. Asterisk denotes significance at the *P* < 0.05 level.

Several additional lines of evidence highlight the unusual nature of these TCO orphan genes. For example, by contrasting the TCO-specific orthologous clusters with the 1:1 TCO-PA42 orthologs, the former are found to have a much higher likelihood of harboring zero to one introns (30% being intron-free), in contrast to the situation for the shared orthologs (only 2% of which are intron-free) (Figure 2A). In one of the largest TCO-specific orthology clusters, 147 genes are located on 106 different scaffolds, with only 19 of them having mRNA support, and 82 having zero and 37 having just one annotated intron, far below the genome-wide average of 5.9 introns/gene in TCO (obtained when these aberrant 7320 genes are excluded). The orphan genes in the TCO-specific clusters are also abnormally short (Figure 2B), have significantly lower GC content (Figure 2C) and higher coverage (Figure 2D) than the 1:1 TCO-PA42 orthologs. Finally, mapping of EST data from TCO (Colbourne *et al.* 2011) shows that only 736 of the 7320 TCO-specific genes have >200 bp covered by such reads; and further results on transcription initiation sites and promoter regions resulting from CAGE data in PA42 (Raborn *et al.* 2016) predict that no more than 143 of these genes are expressed (Table S6 in File S4). These analyses indicate that the excess gene count in the TCO genome is largely a consequence of false positives resulting from overly aggressive gene annotation, while also indicating that the TCO genome harbors a substantial amount of nonfunctional DNA not present in PA42.

**Figure 3:**
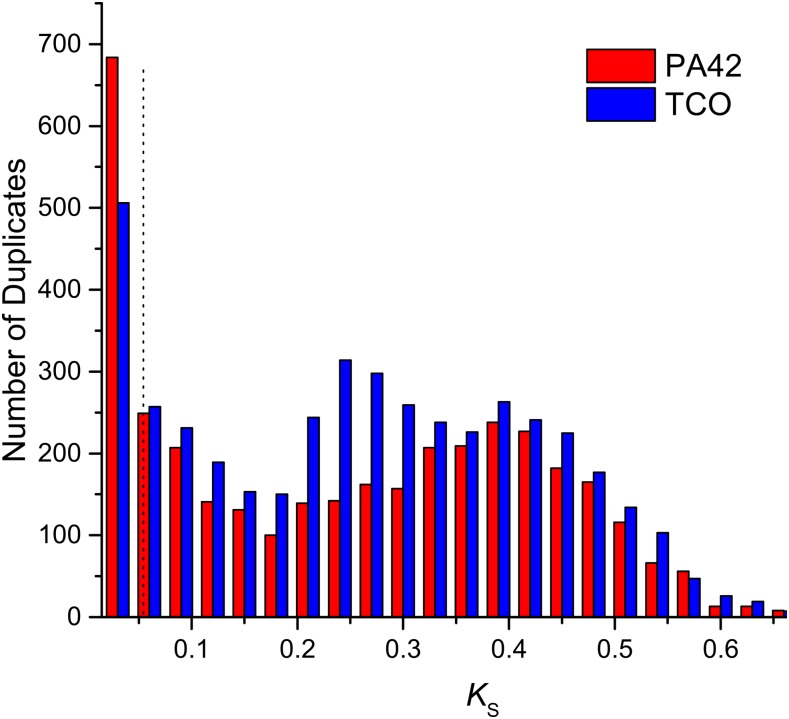
The frequency distribution of *K*_S_ for paralogs within the PA42 genome *vs.* those within TCO. Only genes in orthologous clusters containing both TCO and PA42 genes were used. Sliding-window analyses were used to remove the low quality regions in the alignments, with a cutoff of 0.4 identity for each 15-bp window. The *K*_S_ value for each paralog is the average value when comparing the paralog with others in an orthologous cluster of the genome. The vertical dashed line at 0.057 denotes the average silent-site divergence for pairs of TCO-PA42 orthologs, so that paralogous pairs to the left of this benchmark are younger than the average ortholog divergence between these two clones. *K*_S_, pairwise divergence at silent sites.

Finally, confining our attention to the sets of nonorphan PA42 and TCO genes (*i.e.*, those with orthologs in other metazoan genomes or shared with each other), we evaluated the age distributions of paralogous genes using the magnitude of synonymous-site divergence (corrected for multiple mutations per site) as a measure of age. In TCO, we observed an excess number of duplicate genes having a distance >0.0575 (the average silent-site divergence between orthologs across these two clones) compared to PA42 (Figure 3 and Figure S6 in File S4). If these genes truly exist in TCO, we would expect to see many of them in PA42 due to the fact that they have larger divergence than the orthologs across these two clones. Yet the substantial excess of such duplicate genes annotated in TCO with divergence levels 0.10–0.35 appears to be too high even for sampling error in the latter. This surplus of intermediate-aged duplicate genes in TCO may reflect the fact that they have been lost in PA42, or they may be an artifact of assembly/annotation errors in TCO. On the other hand, the youngest age class of gene duplicates exhibits an excess abundance in PA42.

To determine the impact of annotation errors on the TCO duplicate genes, we extracted all paralogous pairs within TCO and within PA42 with a silent-site distance >0.0575. There are many cases involving such duplicates where PA42 and TCO have the same numbers of copies in the paralogous cluster, and these most likely represent true paralogs between the two clones. After excluding these cases, there are 950 cases of genes in PA42 *vs.* 1724 in TCO with different numbers of copies in the paralogous clusters of annotated genes. In total, only 476 of the 1724 genes in TCO are supported by EST and/or promoter data, whereas in PA42, 944 out of 950 are corroborated by RNA-seq (Table S8 in File S4). This suggests that the annotated paralogous genes in PA42 are most likely correct, while a substantial number of those annotated in TCO may derive from false annotation. For the remaining 1248 (1724−476) paralogous genes within TCO with no transcriptomic support, a substantial proportion appear to be problematic: 320 of them are defined as absent from PA42 due to the lack of homologous DNA sequence, whereas 306 contain premature stop codons and/or have *K*_a_/*K*_s_ ≥ 1.0 in PA42, and 72 do not fit a gene model in PA42.

### Reduced effective population size and genome architecture

As noted above, the TCO source population is thought to have a substantially reduced effective population size relative to populations in the geographic region from which PA42 is derived. If prolonged enough, by increasing the power of genetic drift relative to natural selection, such a shift in the population-genetic environment is expected to enhance the chances of accumulation of mildly deleterious mutations and to facilitate the colonization and/or proliferation of exogenous elements such as TEs, introns, and other genomic insertions that impose slightly deleterious effects (Lynch 2007).

**Figure 4:**
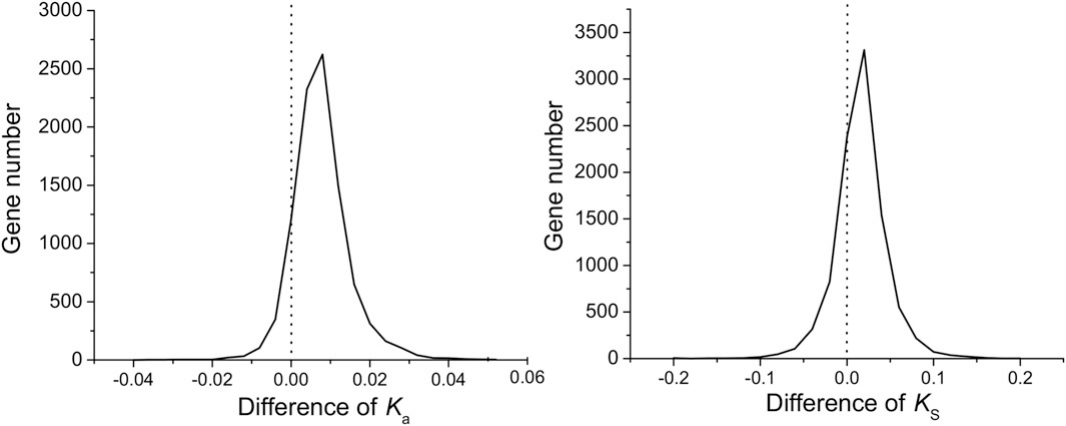
Distributions of the synonymous (*K*_s_) and nonsynonymous (*K*_a_) differences per site per gene in TCO and PA42, using *D. obtusa* as outgroup. *K*_s_ and *K*_a_ were first calculated in TCO *vs.* *D. obtusa* and PA42 *vs.* *D. obtusa*, with the plotted difference providing a measure of the increase in the subtending branch length for TCO above that for the PA42 lineage.

To evaluate the degree to which patterns of molecular evolution differ between the PA42 and TCO genomes, we used the relative-rate test (Sarich and Wilson 1967; Wu and Li 1985) to compare the substitution rates for silent and replacement sites, *K*_s_ and *K*_a_, respectively, using sequence from *D. obtusa* as an outgroup (Tucker *et al.* 2013). The difference between the TCO/*D*. *obtusa* distance and the PA42/*D*. *obtusa* distance provides a measure of the degree of cumulative divergence on the TCO *vs.* the PA42 branch on this three-species tree. Averaging over all compared genes, the *K*_s_ difference is 0.0061 ± 0.0003 (mean ± SE), and the *K*_a_ difference is 0.0057 ± 0.0001. Both *K*_s_ and *K*_a_ are elevated in TCO, while the distribution for *K*_a_ is much more strongly asymmetric toward positive values (Figure 4). Thus, there is either accumulation of deleterious variation or increased substitution rates in TCO.

There are several models showing that effective population size influences the insertion and deletion rates of TEs (Deceliere *et al.* 2005; Le Rouzic *et al.* 2007). In general, larger effective population sizes lead to fewer TEs due to more effective purifying selection. Consistently, more TE copy numbers and higher TE allele frequencies are observed in self-fertilizing *Arabidopsis thaliana* and *Caenorhabditis* nematodes than their outcrossing relatives due to smaller population sizes (Dolgin *et al.* 2008; Lockton and Gaut 2010). To understand whether the same pattern applies to *Daphnia*, we identified the TE content in PA42 using *de novo* searching software of a variety of types (Rho *et al.* 2007; Ellinghaus *et al.* 2008; Rho and Tang 2009), combined with a search for known homologs using RepeatMasker (Smit *et al.* 2004). In total, 530 full-length TEs and 7961 fragmental TEs were identified, constituting ∼7.4% of the assembled PA42 genome (Table 3), and partitioning into 6.6% retrotransposon- and 0.8% DNA transposon-associated DNA. In line with the idea that smaller *N*_e_ facilitates the spread of TEs, PA42 contains significantly less TE-associated DNA content than the 9.4% observed in TCO (*P* < 0.05). To eliminate the impact of genome assemblies on TE contents, we mapped raw reads derived from TCO and PA42 clones to the TE library containing all the TEs from TCO and PA42 (Jiang *et al.* 2017). On average, ∼20% of the reads in clones derived from PA42 mapped to the TEs in the library, whereas ∼33.2% reads from clones derived from TCO mapped to TEs in the library (Table S9 in File S4), indicating more TEs in the TCO genome. This difference is primarily caused by an inflated abundance of LTR retrotransposons in TCO (total genome content of 8.0% *vs.* 5.8% in PA42), which appears to be a product of relatively recent transpositions in TCO. This can be seen by comparing the age distributions of intact LTR elements (determined from the sequence divergence of the flanking LTRs, which are identical at birth), which show an elevation in the youngest class in the TCO genome (Figure S7 in File S4).

**Table 3:** Summary of transposable elements in PA42 3.0

Class	Subclass	Clade	No. of Full-Length (Fragmented) Elements	Fraction of Genome (%)
DNA transposons	TIR	Harbinger	13 (518)	0.23
		Hat	1 (3)	0.01
		p element	3 (10)	0.02
		Mariner	19 (168)	0.15
		isl2eu	50 (172)	0.22
	Helitron	Helitron	26 (215)	0.21
Subtotal			112 (1086)	0.83
Retrotransposons	LTR retrotransposons	Bel	61 (1134)	1.06
		Copia	133 (2408)	2.12
		Gypsy	141 (2059)	2.16
		Dirs	20 (522)	0.47
	Non-LTR retrotransposons	Jockey	18 (246)	0.21
		L1	3 (46)	0.04
		L2	27 (323)	0.31
		Loa	15 (137)	0.14
Subtotal			418 (6875)	6.53
Total			530 (7961)	7.36

TIR, terminal inverted repeat; LTR, long terminal repeat.

*D. pulex* is known to exhibit high levels of intron proliferation, with the discovery of this deriving from observations on the TCO genome (Omilian *et al.* 2008; Li *et al.* 2009, 2014). To determine whether such activity is particularly pronounced in TCO, we conducted a parsimony-based analysis of intron gain and loss following Li *et al.* (2009, 2014), using the flanking exon sequences of predicted introns in both PA42 and TCO to reciprocally blast each other and to query an outgroup species, *D. magna*, to identify intron-free alleles in these assemblies. RNA-seq/EST data were further used to validate the exclusion of introns during transcription.

In total, 50,735 independent intron positions were used in this analysis, and 38,702 were verified using RNA-seq/EST data, among which 128 intron gain/loss events were detected. There were 29 cases in which both PA42 and TCO harbor an intron and *D. magna* does not, and for these cases we were unable to infer whether this represented an intron gain in *D. pulex* or loss in *D. magna*. For the remaining 99 events, we inferred that TCO has experienced 78% more intron gain events than PA42 (57 *vs.* 32; Table 4), as expected if the reduced *N*_e_ of TCO has promoted the accumulation of newly gained introns by random drift (Lynch 2002). Although much less prevalent, intron loss is also overrepresented in TCO (10 *vs.* 0). The lengths of newly gained and lost introns are very similar in both genomes (65–75 bp, Table 4), and very similar to the median intron lengths of 63 and 77 bp in TCO and PA42, respectively.

**Table 4:** Summary of intron gain and loss events in TCO and PA42

	**TCO**	**PA42**
Number of intron gains	57	32
Number of intron losses	10	0
Median length of gained intron (bp)	72	76
Median length of lost intron (bp)	65	N/A
GC composition of gained intron (%)	20	25
GC composition of lost intron (%)	26	N/A

N/A, not applicable.

### Gene family analysis

In the aforementioned analyses using OrthoMCL, 19,269 orthologous gene clusters among PA42, TCO, and the Reference Species were identified and assigned to SCOP families (Murzin *et al.* 1995; Gough *et al.* 2001). In principle, each SCOP family contains protein domains descendent from a common ancestor, and the orthologous clusters containing *Daphnia* genes are present in 2506 such gene families.

**Figure 5:**
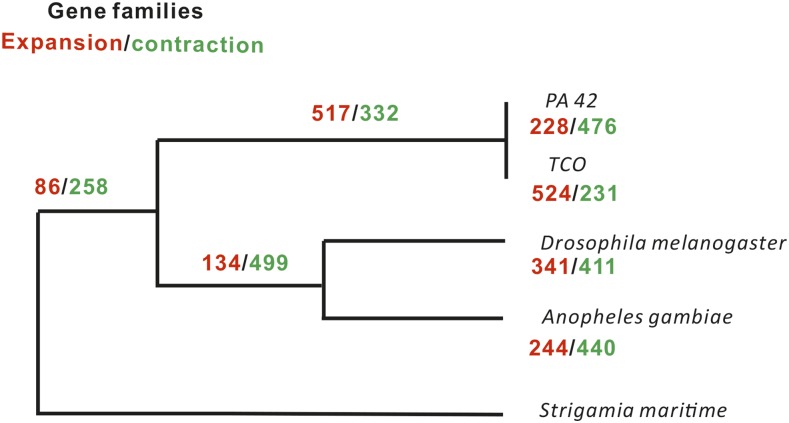
Dynamic evolution of gene families. The gene family expansions and contractions were predicted by CAFÉ 3.0. The species tree required by CAFÉ 3.0 was constructed by 1:1:1 single-copy gene families using the Maximum Likelihood method in MEGA6 (Tamura *et al.* 2013). The RelTime-ML program implemented in the MEGA6 package was used to estimate divergence time among species; calibration time was obtained from the TimeTree database.

The gene family modeling pipeline CAFE 3.0 (Han *et al.* 2013) was used to infer expansion and/or contraction in specific gene families in PA42, TCO, and the Reference Species. Across the phylogeny, most species have higher numbers of gene family contractions than expansions, with TCO and the lineage leading to the common ancestor of TCO and PA42 being the exceptions (Figure 5). This high degree of gene family expansion is consistent with the elevated number of gene gains and retention previously inferred in the TCO genome (Colbourne *et al.* 2011). However, the PA42 genome shows a substantially different pattern, which is much more similar to that in other arthropod lineages. TCO has more gene family expansions and also more genes in a larger portion of families. In 42% of gene families, PA42 and TCO have the same number of genes, but in 40% of the families, the annotated gene number in TCO exceeds that in PA42 (File S2).

Due to the fact that TCO harbors a large number of orphan genes and has an excess of paralogs that may bias the pattern of gene family expansion/contraction, we only focused on the gene family expansion/contraction pattern in PA42. We found 29 gene families to be significantly expanded (*P* < 0.01) in the PA42 lineage (Table S10 in File S4). To further understand the functional enrichment of genes in expanded PA42 gene families, we assigned all of the genes in the expanded PA42 families into functional GO categories. We found that such expansions are mainly enriched in categories such as chitin metabolic process, response to oxidative stress, and protein glycosylation (Table S11 in File S4). We hope that these results will stimulate future research on the role that these gene families play in the functional biology of *D. pulex*.

## Discussion

The development of the first *Daphnia* genome assembly (TCO) greatly advanced *D. pulex* as an important model system for evolutionary, ecological, and environmental studies. The full suite of genomic tools have allowed researchers to investigate the genetic basis of phenotypic traits (Schumpert *et al.* 2015), the origin of obligate parthenogenesis (Xu *et al.* 2015b), environmental sex determination (Kato *et al.* 2011), and genetic response to a wide range of environmental stressors including salinity (Latta *et al.* 2012), toxic cyanobacteria (Asselman *et al.* 2015), and heavy metals (Chen *et al.* 2015). However, previous phylogenetic studies have illuminated the presence of multiple divergent lineages in the *D. pulex* complex (Colbourne *et al.* 1998), and this complicated evolutionary history raises questions about the suitability of the existing TCO genome as a reference for studies involving *D. pulex* isolates sampled outside of OR. Thus, a major motivation of the current study has been to develop a genomic assembly that will facilitate future studies employing animals derived from the geographically widespread lineage *D. pulex*.

To achieve this goal, we established a high quality draft assembly of PA42, a *D. pulex* isolate sampled from a typical woodland pond in the midwest of the USA. We designed our genomic sequencing experiments using short insert-sized libraries and long jumping libraries, which are suitable for *de novo* assembly using AllPathsLG, a leading *de novo* genome assembler using short reads. To reduce the shortcomings of a short read-based assembly, the initial assembly was further improved using 8–10 kb reads generated via Illumina SLRs. Because bacterial DNA contamination can be problematic in the genomic assembly of small organisms, we used independently derived whole-genome sequences and single-sperm sequences of PA42 to remove scaffolds with suspicious contaminant DNA. Finally, because the transcriptome of *Daphnia* might vary substantially between the sexes and under different environmental conditions, to aid in the PA42 gene annotation, we performed RNA-seq for different male and female life stages and in a diverse array of environmental treatments.

Our efforts to acquire an improved *D. pulex* genome sequence resource have proven successful, with the PA42 assembly having a scaffold N50 of 494 kb and 96% of essential eukaryotic single-copy genes appearing in the final product. Although the reduced number of annotated genes in PA42 relative to the earlier derived TCO genome is troublesome, our comprehensive analyses comparing these two draft genomes reveals a number of the sources of erroneous annotation in TCO genome. In TCO, 7320 genes do not have orthologs in PA42: 592 are not covered by any read derived from subsequent TCO mutation-accumulation lines (*i.e.*, appear to be contamination from exogenous DNA), and 2402 are not detected at the DNA level in PA42. Of the remaining TCO-specific genes that do have homologous DNA in PA42, although 85% exhibit >90% sequence identity across the two genomes, none are annotated in PA42; half of them contain premature stop codons, and their average *K*_a_/*K*_s_ is significantly greater than that for 1:1 TCO-PA42 orthologs. In addition to 7320 TCO-specific genes, there are 698 more annotated paralogous genes in TCO relative to PA42, with a silent-site divergence larger than the average divergence of 1:1 TCO-PA42 orthologs and a substantial proportion of these are caused by annotation errors. Therefore, we suggest that due caution should be taken in using the TCO genome annotation in future studies.

We argue that the elevated gene number in TCO mainly comes from aggressive annotation strategies. To support our point of view, we checked the gene number in TCO when applying different annotation strategies. (1) The TCO gene annotation is based on various pieces of evidence including homology, *D. magna*, paralogy, and expressed genes (in total 26,649 genes), and there are 4040 additional genes in TCO without any comparative or empirical support. In contrast, in PA42, we did not use *D. magna* and paralogy as evidence to annotate genes. The quality of the *D. magna* genome is not verified; when contamination existed in *D. magna* genome and was annotated as genes, the same annotation error could be introduced to TCO. The evidence of paralogy should be used with caution, especially in a genome like TCO with a large amount of tandem gene clusters, where alleles could easily be separated and annotated as paralogs. Using the same evidence as we used in PA42, the total gene number in TCO will become 21,361. (2) In TCO, the authors annotated genes with one line of evidence or without any comparative or empirical support; however, in PA42 we were much more conserved and included genes with at least two lines of evidence. When applying our strategy to TCO, the gene number in TCO will be 19,662. If we apply the PA42 annotation strategies in both (1) and (2) to TCO, the total gene number in TCO should be <19,662.

After excluding erroneously annotated genes, there remain >21,000 genes in the TCO genome (File S3), which is still higher than in the PA42 genome. Although further efforts are needed to achieving an accurately annotated TCO genome, we can still learn the potential impact of reduced effective population size on aspects of genomic architecture other than gene number. TE proliferation and intron gain and loss are thought to be strongly affected by effective population size (Lynch 2007). Most TEs are likely to be deleterious across the genome (Tollis and Boissinot 2012; Wei and Cao 2016), but only those with selective disadvantages smaller than the power of random genetic drift are likely to increase in frequency. Consistent with the hypothesis that the TCO genome is contained within a lineage that has experienced a long-term population bottleneck, the proportion of the genome comprising TEs in TCO is higher than that in PA42 (9.40% *vs.* 7.36%). In addition, both the intron gain and loss ratios in TCO are much higher than in PA42, possibly due to the reduced effective population size of TCO promoting the fixation of newly gained introns by random drift (Lynch 2002).

In conclusion, although the TCO genome is certainly of interest from an evolutionary genomics perspective, we argue that it is not a particularly good representative of the *Daphnia* genus, owing to both overly aggressive gene annotation and to evolutionary changes in genomic architectural features (*e.g.*, mobile elements and intron gain/loss) putatively resulting from reduced effective population size. Aside from the annotation issues, the TCO draft genome may still be reasonably suitable for studying the evolution of the *D. pulex* sublineage endemic to OR, whereas the new PA42 genome appears to be a more reliable assembly for examining genomic evolutionary features of *D. pulex* in the remainder of North America.

## Supplementary Material

Supplemental material is available online at www.g3journal.org/lookup/suppl/doi:10.1534/g3.116.038638/-/DC1.
