# Supplementary material for: A New Reference Genome Assembly for the Microcrustacean Daphnia pulex
Source: G3 (Bethesda). 2017 Feb 22;7(5):1405–16. doi: 10.1534/g3.116.038638 (PMC5427498; doi:10.1534/g3.116.038638)
Supplement: Supplementary file 4 [file 1405FileS4.docx]

**Table S1**. DNA libraries used in the assembly. For synthetic long reads, we only used those with length > 8 Kb.

| Type | Insert Size (bp) | Total Reads |
| --- | --- | --- |
| Paired-end | 350 | 187,360 |
| Paired-end | 450 | 47,030,774 |
| Paired-end | 600 | 5,678,346 |
| Mate pair | 3,000 | 13,040,778 |
| Mate pair | 5,000 | 29,897,974 |
| Mate pair | 9,000 | 11,493,406 |
| Mate pair | 19,000 | 14,394,434 |
| Mate pair  Synthetic long reads | 27,000 | 1,609,786  52,443 |

**Table S2.** RNA-seq libraries used for the PA42 annotation.

| Type | Insert Size(bp) |
| --- | --- |
| Life stages | Mature male |
|  | Asexual females |
|  | Sexual females |
| Treatment | 18^o^C |
|  | 26^o^C |
|  | UV light |
|  | Low pH (5.0) |
|  | Nickel (0.03 g/L) |
|  | NaCl (5 g/L)  Atrizine (4 mg/L) |

**Table S3.** Comparison of assembly statistics after connecting scaffolds with synthetic long reads and filtering the genome.

| Type | PA42 1.0^a^ | PA42 2.0^b^ | PA42 3.0^c^ |
| --- | --- | --- | --- |
| Length of scaffolds(bp) | 206,675,209 | 202,316,476 | 156,418,198 |
| Number of scaffolds | 6,173 | 3,312 | 1,822 |
| N50 of scaffolds (bp) | 318,519 | 530,522 | 494,773 |
| L50 of scaffolds | 143 | 107 | 96 |
| Largest scaffold | 3,546,575 | 3,546,408 | 1,661,524 |
| Length of gaps (bp) | 33,808,076 | 27,853,571 | 13,454,790 |

^a^PA42 1.0 is the original assembly from ALLPATH-LG.

^b^PA42 2.0 is the improved version, using synthetic long reads to tie the scaffolds and fill gaps.

^c^PA42 3.0 is the genome filtered from PA42 2.0 by removing potential contaminations.

**Table S4.** The number of gene split events in the TCO and PA42 genomes.

| Split Events | #events |  |
| --- | --- | --- |
| 1 gene in TCO annotated as 2 genes in PA42 | 55 |  |
| 1 gene in TCO annotated as 3 genes in PA42 | 2 |  |
| 1 gene in TCO annotated as 4 genes in PA42 | 1 |  |
| 1 gene in PA42 annotated as 2 genes in TCO | 170 |  |
| 1 gene in PA42 annotated as 3 genes in TCO | 7 |  |
| 1 gene in PA42 annotated as 4 genes in TCO | 2 |  |

**Table S5.** Numbers of gene copies from PA42 and the Reference Species for the orthologous clusters containing the largest numbers of TCO gene annotations. Abbreviations: *Anga, Anopheles gambiae; Stma, Strigamia maritime; Drme, Drosophila melanogaster; Hosa, Homo sapiens; Cele, Caenorhabditis elegans.*

| *Clusters* | *Drme* | *Hosa* | *PA42* | *TCO* | *Stma* | *Cele* | *Anga* |
| --- | --- | --- | --- | --- | --- | --- | --- |
| FLN10 | 0 | 0 | 0 | 147 | 0 | 0 | 0 |
| FLN11 | 0 | 0 | 0 | 132 | 0 | 0 | 0 |
| FLN14 | 0 | 0 | 1 | 119 | 0 | 0 | 0 |
| FLN16 | 0 | 0 | 0 | 111 | 0 | 0 | 0 |
| FLN20 | 1 | 0 | 0 | 97 | 0 | 0 | 0 |
| FLN21 | 0 | 0 | 0 | 96 | 0 | 0 | 0 |
| FLN22 | 0 | 0 | 0 | 91 | 0 | 0 | 0 |
| FLN23 | 0 | 0 | 0 | 91 | 0 | 0 | 0 |
| FLN28 | 0 | 0 | 0 | 86 | 0 | 0 | 0 |
| FLN39 | 0 | 0 | 0 | 75 | 0 | 0 | 0 |
| FLN35 | 0 | 0 | 0 | 62 | 9 | 5 | 0 |
| FLN58 | 0 | 0 | 0 | 58 | 0 | 0 | 0 |
| FLN56 | 0 | 0 | 1 | 58 | 0 | 0 | 0 |
| FLN63 | 0 | 0 | 0 | 54 | 0 | 0 | 0 |
| FLN79 | 0 | 0 | 0 | 48 | 0 | 0 | 0 |
| FLN85 | 0 | 0 | 0 | 46 | 0 | 0 | 0 |
| FLN93 | 0 | 0 | 0 | 45 | 0 | 0 | 0 |
| FLN100 | 0 | 0 | 0 | 43 | 0 | 0 | 0 |
| FLN68 | 1 | 2 | 4 | 42 | 1 | 1 | 1 |
| FLN111 | 0 | 0 | 0 | 40 | 0 | 0 | 0 |
| FLN112 | 0 | 0 | 0 | 39 | 1 | 0 | 0 |
| FLN120 | 0 | 0 | 0 | 38 | 0 | 0 | 0 |

**Table S6**. Analysis of TCO and PA42 lineage-specific gene annotations.

|  | | TCO | PA42 3.0 | |
| --- | --- | --- | --- | --- |
| Number of lineage specific genes: | 7,320 | | | 1,362 |
| Gene missing in alternative genome ^a^ | 2,402 (136 ^b^, 13^c^) | | | 194 (136^d^) |
| No homologous flanking gene ^e^ | 575 (51, 0) | | | 32 (24) |
| One homologous flanking gene | 1,571 (132, 16) | | | 207 (163) |
| Two homologous flanking genes | 2,772 (417, 114) | | | 929 (796) |

^a^ Gene is defined missing if <50% of its DNA sequence can be found in the other genome or its DNA sequence has an identity < 85% with the putatively homologous region in the other genome.

^b^ Numbers indicate TCO-specific genes with a minimum 200 bp TCO EST data support .

^c^ Numbers indicate TCO-specific genes with CAGE (Raborn et al., submitted) peaks within 500 bp of the annotated translation start sites, which provides evidence of transcription initiation.

^d^ Numbers are PA42 genes supported by RNA-seq data with a minimum mapping length of 200 bp.

^e^ Homologous genes are genes in each genome after excluding lineage-specific genes.

**Table S7.** Read mapping frequency of TCO-specific gene clusters and 1:1 TCO-PA42 orthologs. Coding sequences of TCO-specific gene clusters and 1:1 TCO-PA42 orthologs were extracted using perl scripts from TCO genome ([Colbourne et al. 2011](#_ENREF_4)), then reads from each clone were mapped to the coding sequence of each category. Frequency was calculated using the mapped reads divided by the total reads. The data for mutation-accumulation (MA) lines (including TCOMA17, TCOMA237, TCOMA302 and TCOMA97) were obtained from subclones directly derived from TCO (Keith et al. 2016). The data for the *D.* *pulex* clones (including TRO3, JOL2, MORG5, LIS3, 5W2, HUGHES2, STM2, SED2, LYT and GOS1) and OP11 (a clone from another Oregon population) were obtained from Tucker *et al*. (2013). All the raw reads were mapped to TCO genome using BWA default parameter and multiple mapping was randomly assigned a hit.

| Clones | Mapping frequency | |  |
| --- | --- | --- | --- |
|  | TCO-specific genes | TCO orthologs |  |
| TCOMA17 | 0.23 | 0.15 |  |
| TCOMA237 | 0.23 | 0.14 |  |
| TCOMA302 | 0.24 | 0.14 |  |
| TCOMA97 | 0.23 | 0.15 |  |
| OP11 | 0.13 | 0.13 |  |
| TRO3 | 0.11 | 0.15 |  |
| JOL2 | 0.08 | 0.11 |  |
| MORG5 | 0.12 | 0.15 |  |
| LIS3 | 0.12 | 0.16 |  |
| 5W2 | 0.13 | 0.15 |  |
| HUGHES2 | 0.12 | 0.14 |  |
| STM2 | 0.07 | 0.09 |  |
| SED2 | 0.10 | 0.13 |  |
| LYT | 0.13 | 0.16 |  |
| GOS1 | 0.12 | 0.15 |  |
|  |  |  |  |

**Table S8**. Comparison of TCO and PA42 genes in paralogous pairs with *K*_s_ > 0.0575 (the average silent-site divergence between orthologs across these two clones).

|  | | TCO | PA42 3.0 | |
| --- | --- | --- | --- | --- |
| Number of genes: | 1,724 | | | 950 |
| Gene missing in alternative genome ^a^ | 389 (65 ^b^, 12^c^) | | | 111 (109^d^) |
| No homologous flanking gene | 103 (25, 0) | | | 44 (42) |
| One homologous flanking gene | 373 (98, 34) | | | 130 (130) |
| Two homologous flanking genes | 859 (175, 106) | | | 665 (636) |
| Total genes without evidence ^e^ | 1248 | | | 33 |

^a^ Gene is defined missing if <50% of its DNA sequence can be found in the other genome or its DNA sequence has an identity < 50% with the putatively homologous region in the other genome.

^b^ Numbers indicate TCO-specific genes with a minimum 200 bp TCO EST data support.

^c^ Numbers indicate TCO-specific genes with CAGE (Raborn et al., submitted) peaks within 500 bp of the annotated translation start sites, which provides evidence of transcription initiation.

^d^ Numbers are PA42 genes supported by RNA-seq data with a minimum mapping length of 200 bp.

^e^ Numbers are genes without RNA-seq, EST data or CAGE peaks within 500 bp of the annotated translation start sites.

**Table S9.** Summary statistics of read mapped to reference genome and TE library. % stands for the proportion of all the sequencing paired-end reads of each isolate mapped to the object sequences. The data for mutation-accumulation (MA) lines (including TCOMA17, TCOMA237, TCOMA302 and TCOMA97) were obtained from subclones directly derived from TCO (Keith et al. 2016). The data for all the rest clones were obtained from Tucker *et al*. (2013). All the raw reads were mapped to corresponding genome using BWA default parameter and multiple mapping was randomly assigned a hit.

| Clones | Total reads mapped to genome (%) | LTR retrotransposons (%) | Non-LTR retrotransposons (%) | DNA transposons (%) | TE/genome (%) |
| --- | --- | --- | --- | --- | --- |
| 5w-2 | 88.89 | 15.67 | 1.09 | 1.00 | 17.94 |
| jol-2 | 90.57 | 16.40 | 1.15 | 0.99 | 18.90 |
| lis-3 | 93.94 | 16.80 | 1.16 | 0.99 | 19.40 |
| lyt-1 | 93.12 | 17.26 | 1.21 | 0.90 | 19.64 |
| morg-5 | 94.48 | 16.19 | 1.19 | 0.95 | 19.98 |
| sed-2 | 93.39 | 18.65 | 1.26 | 1.03 | 20.17 |
| smi-1 | 88.37 | 16.40 | 1.15 | 0.89 | 20.48 |
| tro-3 | 91.45 | 15.92 | 1.14 | 0.90 | 20.51 |
| hughes2 | 91.13 | 14.47 | 1.02 | 0.86 | 20.79 |
| stm2 | 92.33 | 15.58 | 1.01 | 0.87 | 20.87 |
| eb1 | 93.41 | 16.95 | 1.17 | 1.07 | 20.55 |
| ksp10 | 91.91 | 16.74 | 1.16 | 0.92 | 20.48 |
| lp8b6 | 94.34 | 18.03 | 1.13 | 0.93 | 21.29 |
| nfl3 | 85.59 | 16.03 | 1.05 | 0.92 | 21.03 |
| pa32 | 92.37 | 15.42 | 1.21 | 0.99 | 19.08 |
| povi4 | 89.11 | 16.56 | 1.14 | 0.94 | 20.92 |
| tex9 | 90.60 | 17.53 | 1.18 | 0.95 | 21.70 |
| bw101 | 91.19 | 16.46 | 1.14 | 0.84 | 20.22 |
| tex21 | 94.16 | 15.00 | 1.14 | 0.89 | 18.08 |
| lp8b5 | 92.96 | 14.80 | 1.06 | 0.88 | 18.00 |
| TCOMA17 | 92.15 | 28.39 | 1.19 | 1.27 | 33.48 |
| TCOMA302 | 95.37 | 29.28 | 1.12 | 1.33 | 33.26 |
| TCOMA97 | 94.23 | 28.6 | 1.15 | 1.33 | 32.98 |
| TCOMA237 | 92.53 | 28.37 | 1.11 | 1.31 | 33.27 |

**Table S10.** Significantly (P<0.001) expanded /contracted gene families in PA42. Abbreviations: *Anga, Anopheles gambiae; Drme,Drosophila melanogaster.* Gene umbers beneath each species are the number of genes in a given family.

|  |  |  |  | | | | |  |  |
| --- | --- | --- | --- | --- | --- | --- | --- | --- | --- |
| Name of gene family | | |  | | PA42 | | TCO | Drme | Anga |
| Spermadhesin, CUB domain | | | | Expansion | | 120 | 139 | 30 | 32 |
| FAS1 domain | | | | Expansion | | 25 | 34 | 4 | 5 |
| Fibrinogen C-terminal domain-like | | | | Expansion | | 139 | 99 | 15 | 52 |
| Coagulogen | | | | Expansion | | 46 | 46 | 7 | 7 |
| THAP domain | | | | Expansion | | 50 | 55 | 10 | 16 |
| Myeloperoxidase-like | | | | Expansion | | 54 | 62 | 10 | 19 |
| Pan module (APPLE domain) | | | | Expansion | | 25 | 29 | 5 | 3 |
| FucT-like | | | | Expansion | | 48 | 95 | 4 | 2 |
| Glycosylating toxin catalytic domain-like | | | | Expansion | | 32 | 45 | 2 | 5 |
| YccV-like | | | | Expansion | | 18 | 24 | 1 | 1 |
| Vitelline membrane outer protein-I (VMO-I) | | | | Expansion | | 21 | 8 | 0 | 0 |
| PAPS sulfotransferase | | | | Expansion | | 54 | 63 | 15 | 24 |
| FkbM-like | | | | Expansion | | 13 | 30 | 0 | 0 |
| DNA ligase/mRNA capping enzyme postcatalytic domain | | | | Expansion | | 18 | 16 | 4 | 3 |
| MYND zinc finger | | | | Expansion | | 56 | 62 | 23 | 38 |
| Supernatant protein factor (SPF), C-terminal domain | | | | Expansion | | 31 | 34 | 9 | 7 |
| SRA domain-like | | | | Expansion | | 8 | 6 | 0 | 0 |
| mRNA capping enzyme | | | | Expansion | | 15 | 20 | 3 | 2 |
| Globins | | | | Expansion | | 23 | 26 | 3 | 3 |
| Papain-like | | | | Expansion | | 36 | 56 | 16 | 12 |
| Glycosyl hydrolase family 7 catalytic core | | | | Expansion | | 7 | 7 | 0 | 0 |
| Hypoxia-inducible factor HIF inhibitor (FIH1) | | | | Expansion | | 17 | 23 | 10 | 7 |
| beta 1,4 galactosyltransferase (b4GalT1) | | | | Expansion | | 20 | 15 | 6 | 4 |
| Acetylhydrolase | | | | Expansion | | 12 | 12 | 1 | 3 |
| Tudor domain | | | | Expansion | | 47 | 62 | 24 | 25 |
| 28-residue LRR | | | | Expansion | | 68 | 71 | 28 | 30 |
| Matrix metalloproteases, catalytic domain | | | | Expansion | | 19 | 13 | 3 | 3 |
| Astacin | | | | Expansion | | 31 | 34 | 13 | 13 |
| Lipovitellin-phosvitin complex; beta-sheet shell regions | | | | Expansion | | 17 | 23 | 4 | 9 |

**Table S11.** Gene Ontology enrichment analysis for expansion of specific gene families in PA42 genome compared to the background expansion rate. For each GO term 2x2 contingency table were used to record the number of genes included or not included for expanded gene families (group1), we also recorded the total number of genes in and out of the specific GO (group 2), andχ^2^ test were used to calculate the P-value of group1 and group2.

| GO ID |  | | GO Name | No.Genes | P value |
| --- | --- | --- | --- | --- | --- |
| GO:0006030 | BP | chitin metabolic process | | 91 | 0 |
| GO:0006979 | BP | response to oxidative stress | | 53 | 3.25E-72 |
| GO:0033578 | BP | protein glycosylation | | 48 | 4.61E-38 |
| GO:0030198 | BP | extracellular matrix organization | | 20 | 1.54E-31 |
| GO:0006370 | BP | 7-methylguanosine mRNA capping | | 18 | 2.61E-30 |
| GO:0006508 | BP | proteolysis | | 103 | 2.58E-28 |
| GO:0048477 | BP | oogenesis | | 10 | 1.06E-16 |
| GO:0015671 | BP | oxygen transport | | 8 | 1.86E-14 |
| GO:0006032 | BP | chitin catabolic process | | 14 | 2.97E-14 |
| GO:0016998 | BP | cell wall macromolecule catabolic process | | 14 | 7.66E-14 |
| GO:0007155 | BP | cell adhesion | | 20 | 3.09E-13 |
| GO:0006629 | BP | lipid metabolic process | | 13 | 1.23E-06 |
| GO:0055114 | BP | oxidation-reduction process | | 56 | 1.56E-06 |
| GO:0005975 | BP | carbohydrate metabolic process | | 26 | 5.62E-06 |
| GO:0006355 | BP | regulation of transcription, DNA-templated | | 8 | 8.78E-06 |
| GO:0006687 | BP | glycosphingolipid metabolic process | | 6 | 2.27E-05 |
| GO:0072668 | BP | tubulin complex biogenesis | | 7 | 2.86E-05 |
| GO:0034333 | BP | adherens junction assembly | | 7 | 4.65E-05 |
| GO:0070830 | BP | bicellular tight junction assembly | | 7 | 4.65E-05 |
| GO:0009058 | BP | biosynthetic process | | 6 | 3.08E-04 |
| GO:0000226 | BP | microtubule cytoskeleton organization | | 7 | 6.70E-04 |
| GO:0055085 | BP | transmembrane transport | | 7 | 7.35E-03 |
| GO:0043547 | BP | positive regulation of GTPase activity | | 7 | 2.33E-02 |
| GO:0004601 | MF | peroxidase activity | | 53 | 2.28E-83 |
| GO:0005201 | MF | extracellular matrix structural constituent | | 60 | 1.37E-81 |
| GO:0008417 | MF | fucosyltransferase activity | | 48 | 1.13E-69 |
| GO:0020037 | MF | heme binding | | 75 | 1.02E-63 |
| GO:0008146 | MF | sulfotransferase activity | | 40 | 5.85E-57 |
| GO:0008234 | MF | cysteine-type peptidase activity | | 34 | 2.05E-50 |
| GO:0004222 | MF | metalloendopeptidase activity | | 52 | 1.77E-49 |
| GO:0019825 | MF | oxygen binding | | 22 | 1.19E-34 |
| GO:0050839 | MF | cell adhesion molecule binding | | 20 | 1.54E-31 |
| GO:0004484 | MF | mRNA guanylyltransferase activity | | 17 | 9.67E-29 |
| GO:0005319 | MF | lipid transporter activity | | 17 | 2.17E-24 |
| GO:0042393 | MF | histone binding | | 8 | 1.19E-12 |
| GO:0005524 | MF | ATP binding | | 6 | 7.32E-12 |
| GO:0001733 | MF | galactosylceramide sulfotransferase activity | | 6 | 3.15E-11 |
| GO:0003676 | MF | nucleic acid binding | | 70 | 4.58E-07 |
| GO:0004869 | MF | cysteine-type endopeptidase inhibitor activity | | 5 | 3.55E-06 |
| GO:0046872 | MF | metal ion binding | | 46 | 1.59E-05 |
| GO:0048487 | MF | beta-tubulin binding | | 7 | 4.65E-05 |
| GO:0005044 | MF | scavenger receptor activity | | 5 | 4.92E-05 |
| GO:0008270 | MF | zinc ion binding | | 66 | 1.13E-03 |
| GO:0016810 | MF | hydrolase activity, acting on carbon-nitrogen (but not peptide) bonds | | 5 | 1.92E-03 |
| GO:0004553 | MF | hydrolase activity, hydrolyzing O-glycosyl compounds | | 7 | 3.05E-02 |
| GO:0005578 | CC | proteinaceous extracellular matrix | | 80 | 0 |
| [GO](http://www.ebi.ac.uk/QuickGO/GTerm?id=GO:0005581):0005581 | CC | collagen trimer | | 60 | 1.37E-81 |
| GO:0042600 | CC | chorion | | 21 | 5.41E-35 |
| GO:0031012 | CC | extracellular matrix | | 18 | 8.67E-24 |
| GO:0005833 | CC | hemoglobin complex | | 8 | 1.86E-14 |
| GO:0005615 | CC | extracellular space | | 20 | 9.95E-10 |
| GO:0016021 | CC | integral component of membrane | | 16 | 4.96E-08 |
| GO:0005923 | CC | bicellular tight junction | | 7 | 2.86E-05 |
| GO:0016328 | CC | lateral plasma membrane | | 7 | 2.86E-05 |
| GO:0005794 | CC | Golgi apparatus | | 6 | 4.18E-05 |
| GO:0005912 | CC | adherens junction | | 7 | 4.65E-05 |
| GO:0016020 | CC | membrane | | 66 | 1.35E-03 |
| GO:0005737 | CC | cytoplasm | | 5 | 1.98E-02 |

BP: Biological process; MF: Molecular function; CC: Cell component


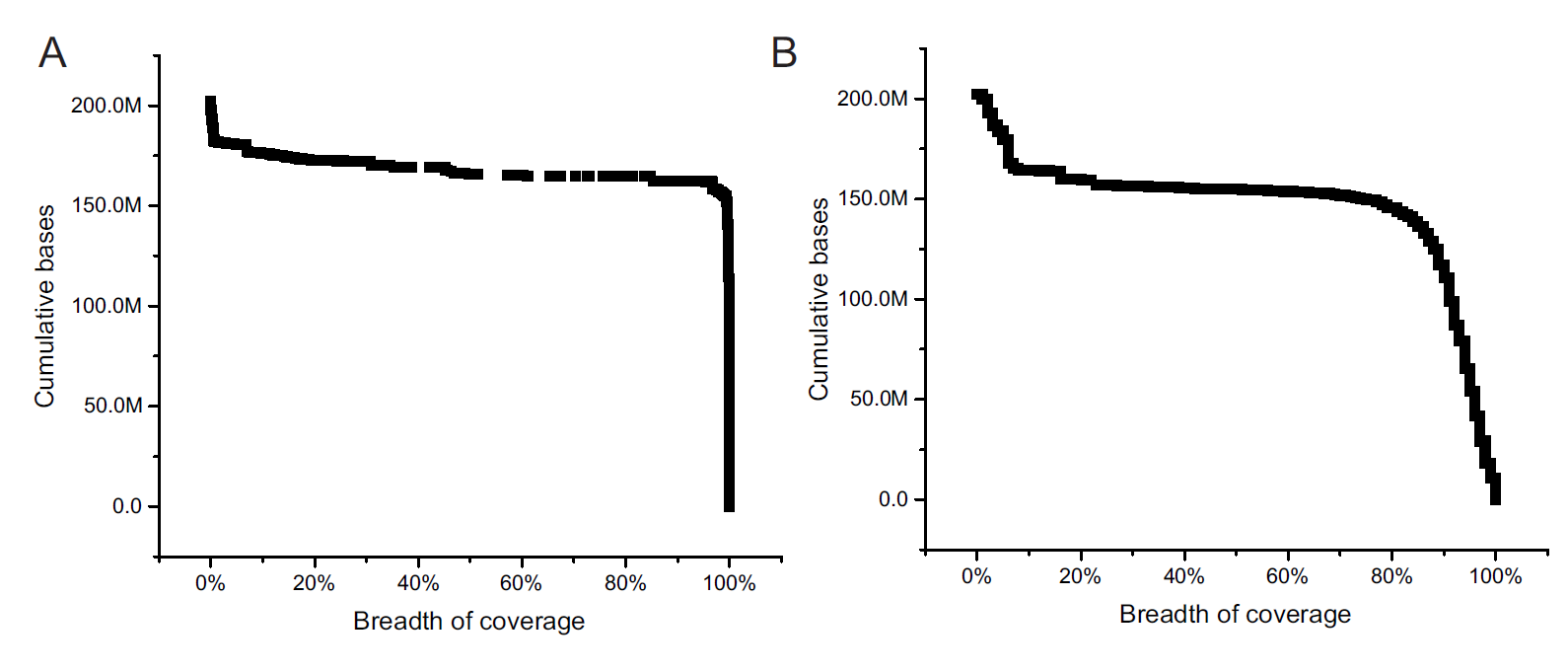


**Figure S1.** The graphic shows the relationship of cumulative nucleotide bases of scaffolds with the breadth of coverage (bases in a scaffold were covered by sequenced reads) for each scaffolds. The breadth of coverage was calculated for each scaffold by mapping the 200 million independent paired-end reads (PE reads) and sperm reads to the assembly of PA42 2.0 using bwa-mem (version 0.7.2). Then we sum the total length of scaffolds at each coverage level as the cumulative bases. (A) Breadth of coverage and cumulative bases (y-axis) of scaffolds when mapping the PE reads to the assembly PA42 2.0. (B) The breadth of coverage and cumulative bases (y-axis) of scaffolds when mapping the sperm reads to the assembly PA42 2.0.


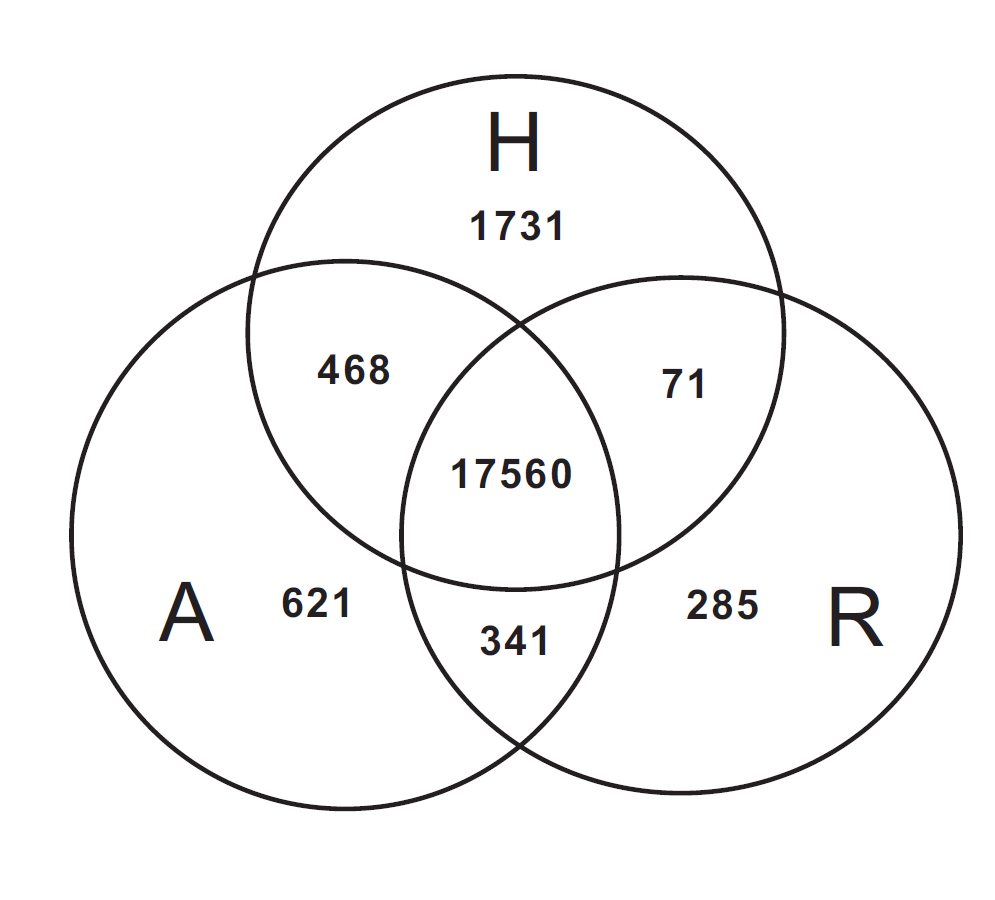


**Figure S2.** Summary of gene models derived from three types of evidence (H: protein homolog, R: RNA-seq, A: *ab initio* prediction). Proteins from the genomes of five reference metazoans (*S. maritime, A. gambiae, C. elegans, D. melanogaster,* and *H. sapiens*) together with 458 proteins from CEGMA (Parra et al. 2007) were used as input for MAKER (Holt and Yandell 2011) to identify genes with multiple lines of evidence. A total of 177,598 transcripts generated by assembly RNA-seq from three different life stages and seven different environmental conditions were used in these analyses. 800 genes with complete gene structure (i.e., possessing 3ʹ UTR, 5ʹUTR, and > 3 exons) supported by RNA-seq evidence were used to train gene models for *ab initio* prediction.


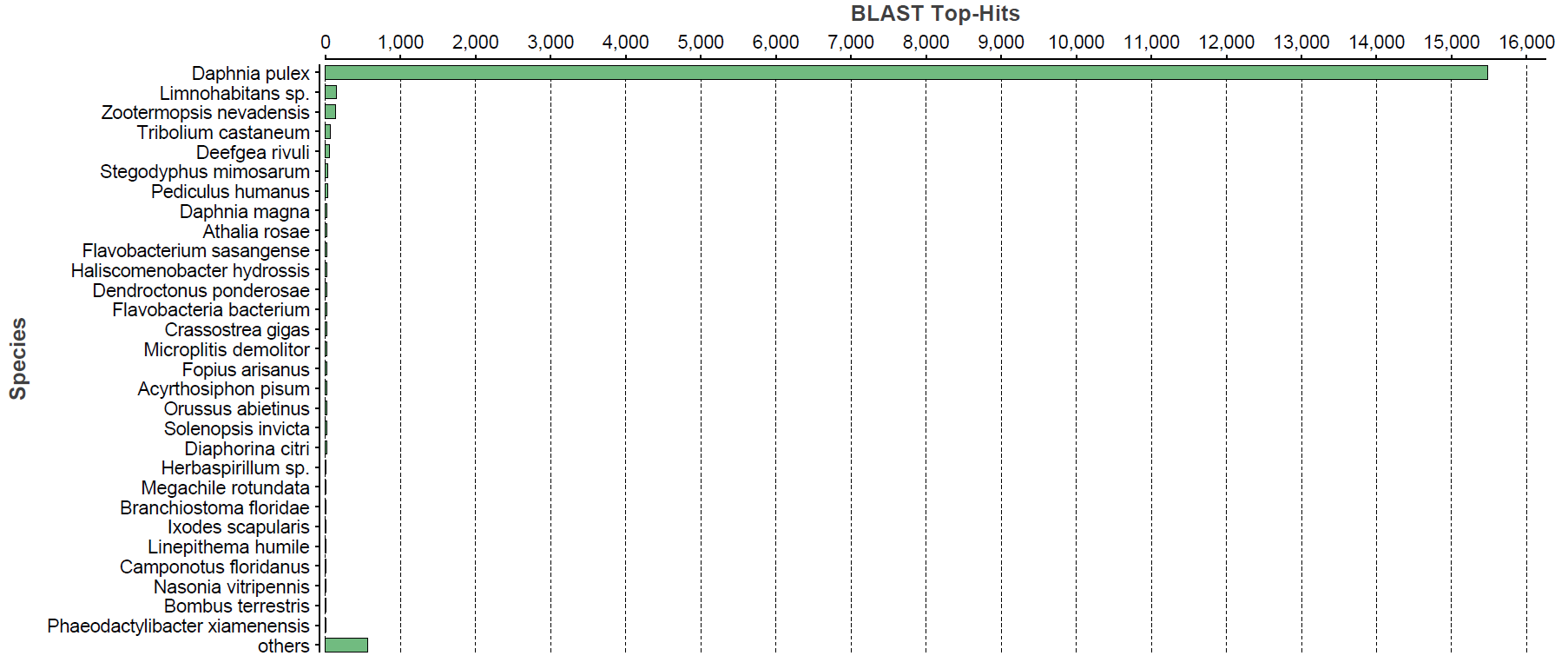


**Figure S3.** Top-hit species distribution for predicted PA42 genes, obtained by querying (with Blast) the PA42 gene models against the non-redundant database in NCBI.


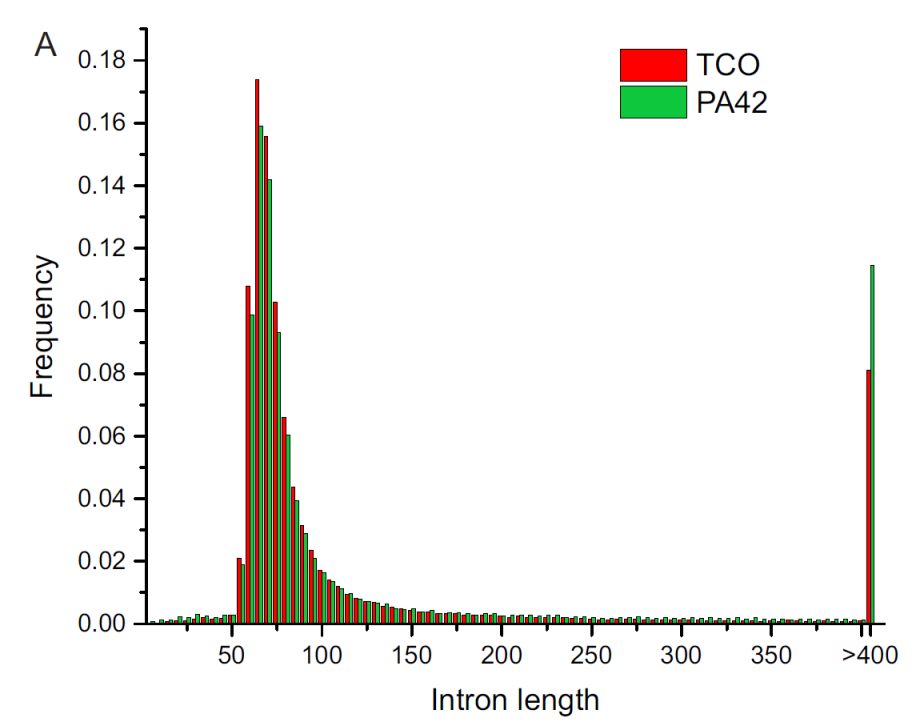


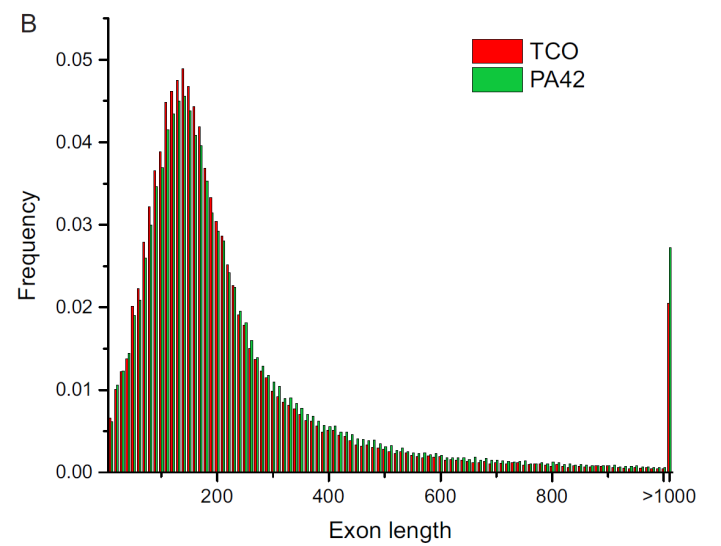


**Figure S4.** Frequency distributions of intron (A) and exon (B) lengths in PA42 and TCO.


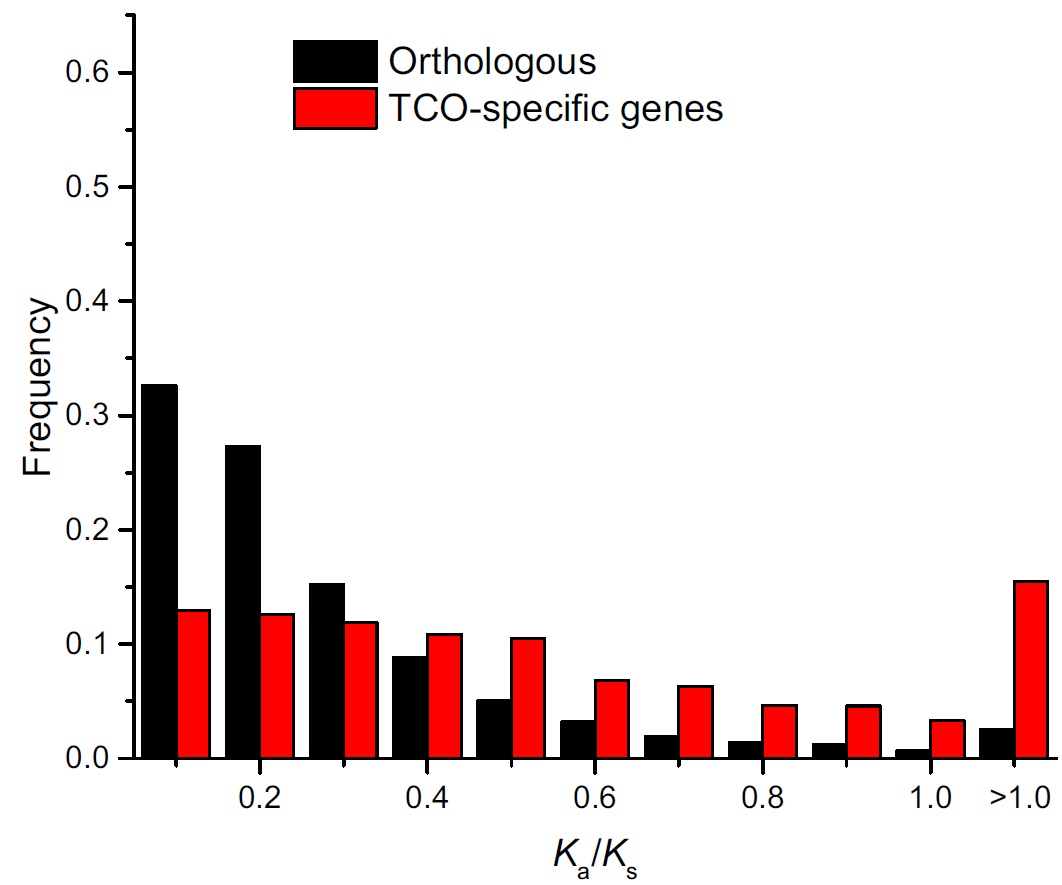


**Figure S5.** Distribution of *K*_a_/*K*_s_ for 1:1 PA42 TCO orthologs, contrasted against that for annotated TCO-specific genes with homologous DNA sequences (but unannotated) in the PA42 genome.


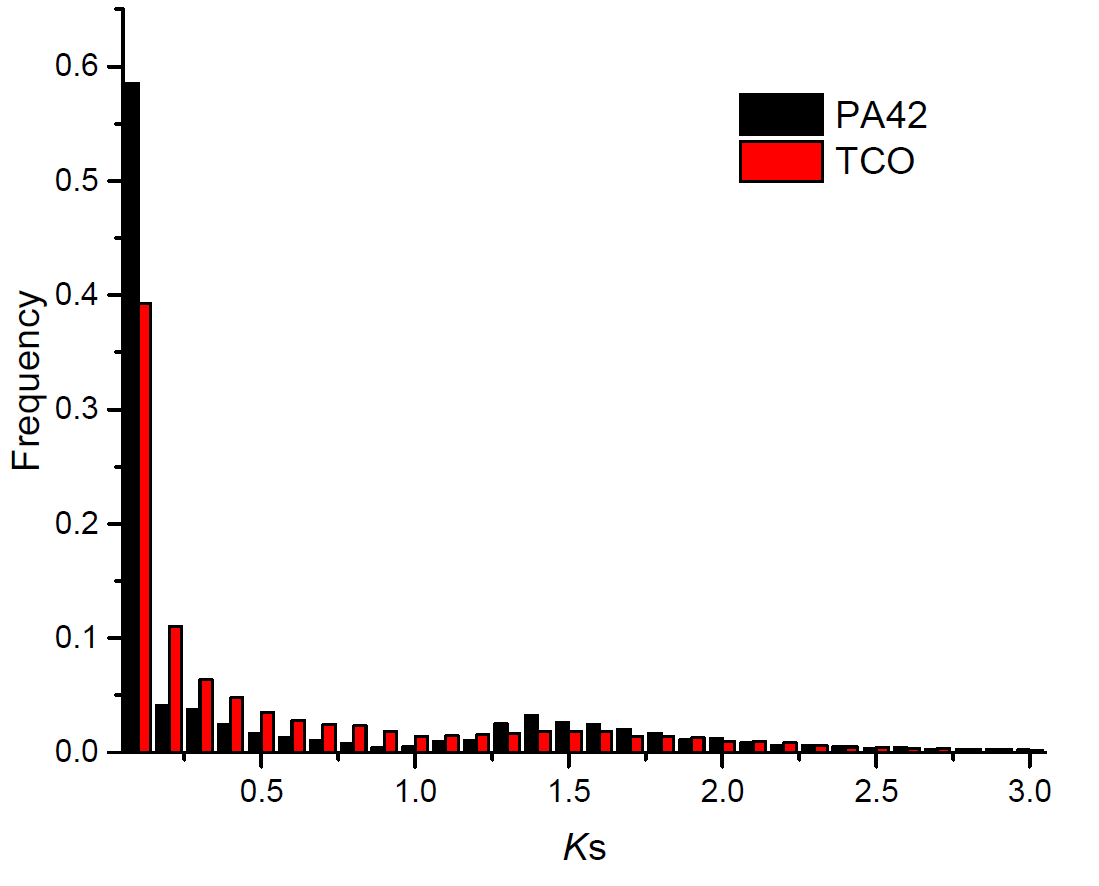


**Figure S6.** Frequency of pair-wise genetic divergence at silent sites (*K*s) among all gene duplicates in the PA42 and TCO, for genes with >100 aligned amino acids and percent identity >80% (5,377and 28,772 pair-wise comparisons for the two genomes, respectively).


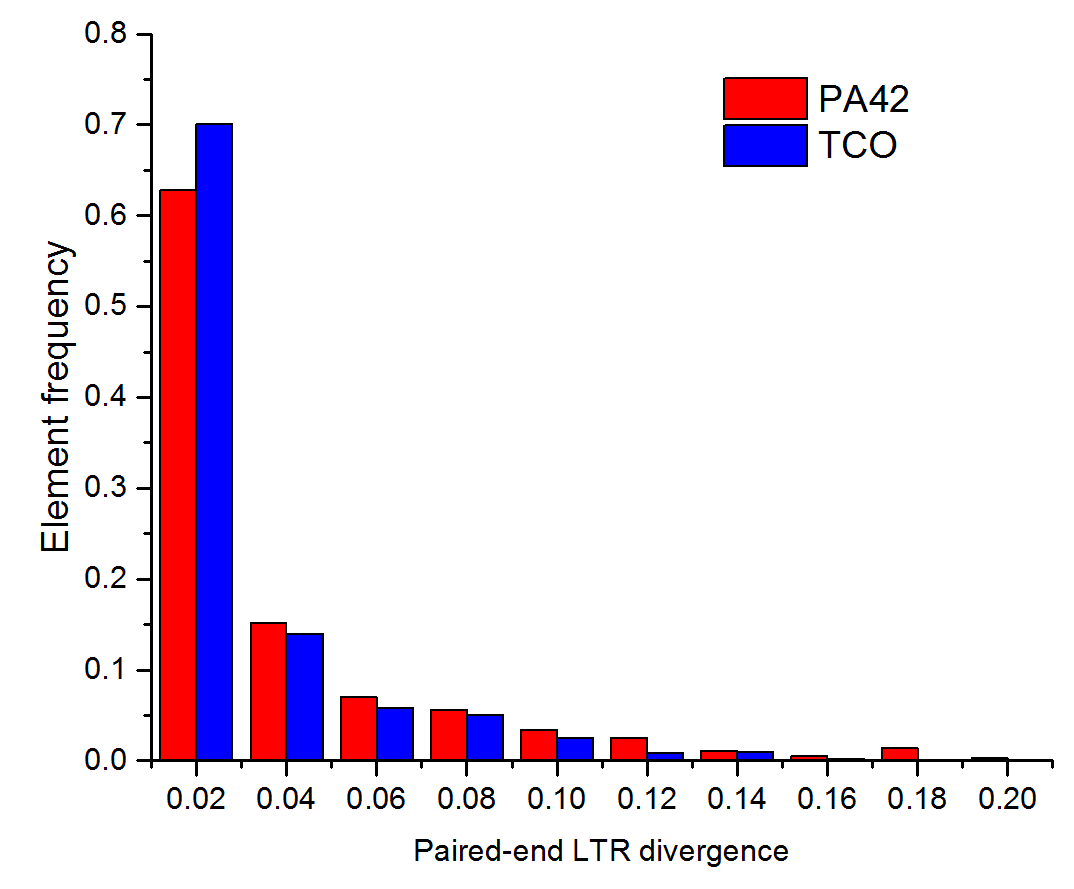


**Figure S7.** The frequency distribution of LTR divergence in PA42 vs. TCO, in units of substitutions per site between flanking LTR sequences.

**References**

Holt C, Yandell M. 2011. MAKER2: an annotation pipeline and genome-database management tool for second-generation genome projects. *BMC Bioinformatics* **12**: 491.

Parra G, Bradnam K, Korf I. 2007. CEGMA: a pipeline to accurately annotate core genes in eukaryotic genomes. *Bioinformatics* **23**:1061-1067.
